# The Functional Role of Fungi and Bacteria in Sulfur Cycling During Kelp (*Ecklonia Radiata*) Degradation: Unconventional Use of PiCrust2

**DOI:** 10.1111/1758-2229.70140

**Published:** 2025-07-24

**Authors:** Anita K. Perkins, Hans‐Peter Grossart, Keilor Rojas‐Jimenez, Alice Retter, Joanne M. Oakes

**Affiliations:** ^1^ Aquatic Botany and Microbial Ecology Research Group HUN‐REN Balaton Limnological Research Institute Tihany Hungary; ^2^ National Laboratory for Water Science and Water Security HUN‐REN Balaton Limnological Research Institute Tihany Hungary; ^3^ Centre for Coastal Biogeochemistry Faculty of Science and Engineering, Southern Cross University Lismore New South Wales Australia; ^4^ Leibniz Institute for Freshwater Ecology and Inland Fisheries (IGB), Experimental Limnology Neuglobsow Germany; ^5^ University of Potsdam Institute of Biochemistry and Biology Potsdam Germany; ^6^ Escuela de Biologia, Universidad de Costa Rica San Jose Costa Rica

**Keywords:** bacteria‐fungi interactions, carbon cycling, filamentous fungi, functional roles, kelp remineralization, metabolic pathways, sulfur cycling

## Abstract

Macroalgae is a major source of detritus in coastal ecosystems, contributing approximately 1521 ± 732 Tg C year^−1^ to global net primary production. Fungal remineralisation of *Ecklonia radiata* detritus produces substantial amounts of dimethylsulfoniopropionate, total alkalinity, and dissolved inorganic carbon, supporting coastal biogeochemical cycles. To expand on the role of fungi during *E. radiata* degradation, we examined changes in fungal and bacterial communities at the start and after 21 days in a mesocosm, comparing microbial functional roles between blades and stipes. We employed next‐generation sequencing to evaluate the potential contributions of fungi and bacteria, and additionally utilized FUNGuild, FungalTraits, and PiCrust2 databases. We cross‐referenced the metabolic pathways predicted by PiCrust2 with the literature to determine whether these pathways have been documented in fungi. Of the 423 metabolic pathways identified, 342 have also been reported in fungi, including 281 redox‐related pathways, 220 associated with nicotinamide adenine dinucleotide, and 194 linked to sulfur metabolism. These overlaps suggest that bacteria and fungi could play complementary roles in kelp degradation, contributing distinct yet interconnected functions. Our results highlight that these metabolic pathways cannot be attributed to bacteria alone and fungi are essential to kelp remineralisation.

## Graphical abstract

The microbial remineralization of *Ecklonia radiata* produces substantial amounts of climate‐relevant compounds, including dimethylsulfoniopropionate, carbon, sulphur and alkalinity. While bacterial metabolic pathways are well‐characterised and relatively predictable, fungal contributions remain underexplored due to the lack of metabolic predictive tools like Picrust. Using bacterial data, this study identifies overlapping functional pathways, suggesting that fungi may also play a key role in *E. radiata* remineralisation through NAD ^+^‐dependent and redox‐related processes. These fungal‐mediated processes are likely important contributors to coastal biogeochemical cycles.

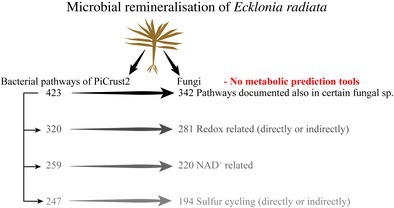

## 1 Introduction

Macroalgae are highly productive ecosystems, occupying approximately 23% of the world's coastlines (Krause‐Jensen and Duarte 2016). The estimated macroalgal net primary production (NPP) is approximately 1521 Tg C year^−1^ (ranging from 1020 to 1960 Tg C year^−1^) of which about 58% is rapidly remineralised around the coastal shelf (Krause‐Jensen and Duarte 2016). Since remineralisation drives biogeochemical cycles (Nicholls et al. 2016), macroalgal degradation plays a significant role in coastal carbon and sulphur cycling (Hill et al. 2015; Raven 2018). By driving these cycles, macroalgal remineralisation influences climatic feedback and connects to broader marine biogeochemical processes (Perkins et al. 2023). The activity of fungi and bacteria mediates remineralisation, and these microorganisms are driving the processing of this vast kelp biomass (Perkins et al. 2023; Perkins et al. 2021). Therefore, understanding the interactions between fungi and bacteria during kelp degradation and remineralisation is essential for a deeper insight into marine carbon and sulphur cycling.

Most studies on kelp degradation (Kessler et al. 2018; Stratil et al. 2013; Bengtsson 2011; Bengtsson and Øvreås 2010; Bengtsson et al. 2011) have focused on bacterial contributions, with only a limited number addressing the role of fungi (Perkins et al. 2023; Perkins et al. 2021), despite the growing recognition of fungi's metabolic versatility and capabilities. Studies on kelp degradation have shown that bacteria are responsible for aerobic degradation, fermentation, and sulfur cycling (Van Erk et al. 2020). Additional research has examined bacterial‐driven polysaccharide degradation (Brunet et al. 2021), the bacterial processes that release dissolved organic carbon (DOC) during kelp degradation (de Bettignies et al. 2020), which then enhances carbon sequestration through the formation of refractory DOC (Zhang et al. 2022). However, this bacterial‐centric focus has largely overlooked fungi, potentially leading to misinterpretation or an incomplete understanding of microbial dynamics and their broader impacts on marine carbon or sulfur cycling.

One study highlighted the remarkable role of fungi in kelp degradation, substantially increasing the production of dissolved inorganic carbon (DIC), total alkalinity (TA), and dimethyl sulfoniopropionate (DMSP) (Perkins et al. 2023). Another study has found that DMSP and proline prevent bacterial attachment to *Fucus vesiculosus* (Saha et al. 2012). Fungal reactive enzymes, such as peroxidases (Perkins et al. 2023) (well‐documented in terrestrial environments (Conesa et al. 2002)), may play an important role in modifying kelp‐associated compounds like phlorotannins (de Bettignies et al. 2020), which may influence sulfate oxidation/reduction and the production of compounds such as DMSP. These findings demonstrate how fungal metabolites can shape microbial interaction during degradation and remineralisation, thus regulating bacterial activity (Saha et al. 2012; Gahan and Schmalenberger 2014; Gahan et al. 2022).

Sulfur cycling is fundamental to numerous microbial processes such as enzyme function, cellular respiration, and protein synthesis, while also linking carbon cycling to climate regulation (Mukwevho et al. 2014; Li et al. 2018; Hines 1996). Although bacterial sulfur‐reducing and oxidising capabilities (Mußmann et al. 2007; Imhoff 2020; Elshahed et al. 2003), and their associated genes (Mußmann et al. 2007; Baker et al. 2015; Hu et al. 2018), have been extensively studied, fungal utilisation of unique intermediates and pathways remains underexplored (Linder 2012, 2018; Marzluf 1993). Fungi, such as *Aspergillus* sp., synthesise ergothioneine (EGT) and produce amino acids like cysteine and methionine, crucial for antioxidant production and protein synthesis, as well as defence compounds such as S‐adenosylmethionine (SAM), S‐adenosylhomocysteine (SAH), and gliotoxin (Paietta 2004; Traynor et al. 2019). In addition, fungi demonstrate notable metabolic versatility in assimilating various sulfur sources (e.g., sulfides, sulfoxides, and sulfate esters (Linder 2012; Linder 2018)) and demonstrate distinct responses to sulfur limitation (Paietta 2004). Since sulfur is a critical element in kelp degradation, bacterial contributions to these processes have been investigated (Van Erk et al. 2020; Brunet et al. 2021; Zhang et al. 2023), while only one study explored the potential role of fungi (Perkins et al. 2023). In this study, both fungal and bacterial potential contributions were explored, and such comparisons are essential to define the respective roles of bacteria and fungi.

Here, our primary objective was to simultaneously assess fungal and bacterial communities during the degradation of *E. radiata* tissues (blade and stipe) over 21 days, simulating beach conditions in a mesocosm setup. Microbial community composition and functional roles were determined using next‐generation sequencing (NGS) and bioinformatics tools (FUNGuild, FungalTraits, and PiCrust2). FUNGuild assigns ecological roles to fungi, FungalTraits provides trait‐based insights into fungal species, and PiCrust2 predicts metabolic pathways from bacterial sequencing data. Since no tool equivalent to PiCrust2 exists for fungi, we examined the metabolic pathways predicted by PiCrust2 to determine if these pathways are also documented for fungi. We primarily assessed these pathways to (1) identify any links to sulfur processing and (2) uncover any documented fungal involvement by searching the MetaCyc database and Google Scholar for relevant metabolic studies on fungi. While this approach is unconventional and may invite scepticism, fungal metabolism and their ecological impacts are important to provide deeper insights into microbial biogeochemical cycling in aquatic systems and elsewhere.

## 2 Materials and Methods

### 2.1 Collection of Experimental Material

Three whole healthy kelp specimens (*Ecklonia radiata*) with no visible disease were collected via SCUBA diving at Charlesworth Bay, Solitary Islands Marine Park (NSW, Australia, 30°16′3.8″ S, 153°8′40.2″ E) in June 2022. The kelp was transported to the laboratory in fresh seawater and stored for ~6 h before incubation commenced. Surface sand was collected during low tide from Angel's Beach, Ballina (2°51′6.385″ S, 152°36′2.396″ E). The sand was sieved to remove any organic material or beach cast. Fresh seawater was collected weekly from Angel's Beach, Ballina (NSW, Australia, 2°51′6.385″ S, 152°36′2.396″ E) at high tide. The seawater was stored outdoors in shade‐cloth‐covered 20 L containers until use and continuously bubbled with air to maintain oxygen levels and freshness.

### 2.2 Experimental Setup

Eight clear Plexiglas core liners (50 cm long × 9 cm diameter tubes) were used for the incubations to simulate beach conditions (Figure S1). The lower end of each core liner was capped with a Plexiglass plate that tapered to a single 3 cm diameter outlet. This outlet was covered with a 100 μm nylon mesh, which retained sand and allowed water to drain from the cores. To prevent temperature fluctuations and photo‐oxidation within subsurface sediments, each mesocosm was enveloped in aluminium foil and placed within a frame wrapped in shade cloth that shaded the cores to ~80% of their height. The choice of a 21‐day duration was based on our earlier results (Perkins et al. 2023, 2022), which consistently showed high levels of carbon and sulphur at this stage of the degradation.

At the beginning of the incubations, homogenised sand was placed into each core liner to a depth of 8 cm, after wetting to settle the sand. The kelp parts were separated into blade and stipe, as their structural differences (Trevathan‐Tackett et al. 2015; Barbosa et al. 2020; Kaidi et al. 2022) may support distinct microbial communities and degradation dynamics. Kelp stipes were cut into 3–5 cm lengths with a sterilised scalpel (70% ethanol); a section of stipe material was added to each of the three cores (three replicates), and the remaining portions were stored in 70% ethanol (in sterile 50 mL Falcon tubes) for microbial analysis (see below). For blades, lateral sections were sliced in half lengthwise; one half was added to the cores (three replicates), and the other half was placed in 70% ethanol. The eight cores were arranged as follows: two cores contained only sand and served as controls with no kelp detritus added, three cores included blade material, and three cores included stipe material. To sufficiently bury the kelp, the cores (including controls) were filled with additional sand to a total depth of 48 cm.

Cores were incubated outdoors in direct sunlight for 21 days. Each day, at 9 am and 5 pm, 500 mL of seawater was gently added to the surface of each core and immediately allowed to drain, imitating the tidal re‐wetting of buried kelp detritus in the upper tidal zone. During each tidal simulation, the water permeated through the sediment and drained away through the base hole. Following drainage, the water level was maintained just beneath the kelp.

### 2.3 Sample Collection for Microbial Analysis

To assess microbial community dynamics over time, samples were analysed as follows: blade and stipe material from the start (S) of the incubation (pre‐burial) (day 0; “BladeS” and “StipeS”); and all other samples collected at the end of the 21‐day incubation, including blade and stipe material (“BladeE” and “StipeE”), sand from control mesocosms (“Control”/“Sand”), and sand collected adjacent to remaining blade or stipe material (“Sand + Blade” and “Sand + Stipe”). Comparison of samples from “Sand” with “Sand + Blade” and “Sand + Stipe” aimed to distinguish microbial communities that developed due to the presence of kelp material from those that would otherwise be present in the surrounding sediment. Upon completion of the 21‐day incubation, the bottom of each core was removed, and the upper 37 cm of sand was extruded. Additional sand was then carefully removed until kelp detritus remnants were identified, or, for control cores, until a depth equivalent of ~40 cm (i.e., the depth at which detritus was buried in treatment cores) was reached. A homogenised sample of sand was collected from this depth in all cores, immediately adjacent to any remaining detritus in treatment cores. Sand samples were transferred into sterile 50 mL Falcon tubes and stored frozen (−80°C) until further analysis. For cores containing kelp detritus, the remaining sand within each core was gently passed through a 1 mm mesh sieve sterilised with 70% ethanol. Fragments of detritus retained on the sieve were transferred to sterile 50 mL Falcon tubes using sterile tweezers (70% ethanol) and preserved in 70% ethanol. For detailed microbial processing, see Supporting Information.

### 2.4 Bioinformatics Analyses

We used the DADA2 (v1.21) in R (v4.1.2, R Core Team 2022) to process the Illumina‐sequenced paired‐end fastq files to generate an amplicon sequence variants (ASVs) table with higher‐resolution analogs than the traditional OTUs (Callahan et al. 2016). The pipeline parameters for filtering and trimming were set as TruncLen = c (240, 160), MaxN = 0, MaxEE = c (2) to improve quality control. Briefly, we removed primers and adapters, inspected the quality profiles of the reads, filtered and trimmed sequences with a quality score < 30, estimated error rates, modelled and corrected for amplicon errors, and inferred the sequence variants. Then, we merged the forward and reverse reads to obtain the full denoised sequences, removed chimeras, and constructed the ASV table.

Taxonomy was assigned using the function assignTaxonomy of DADA2 (Callahan et al. 2016). For 16S rRNA sequences, the SILVA database (v.138.1)—a curated non‐redundant small‐subunit (SSU)—was used (Quast et al. 2013; Glöckner 2019). To improve the taxonomic resolution, a second classification was performed with the IDTAXA classifier from the DECIPHER package with both the SILVA v.138.1 and RDP database v.18 (http://rdp.cme.msu.edu/) (Murali et al. 2018). The secondary classification aimed to increase confidence in taxonomic assignments, particularly for underrepresented environmental sequences. For fungal ITS rRNA sequences, taxonomy was also assigned using DADA2 with the UNITE database v.10 (Abarenkov et al. 2021). The consistency between the taxonomic assignments from different programmes was verified through additional manual curation. In cases of discrepancies, sequences were compared using BLASTn against the NCBI Genbank database, and, where possible, taxonomic assignments were accepted if the top hit showed a percent identity of ≥ 98.5% and an *E*‐value ≤ 1e–80. For the 16S rRNA dataset, sequences assigned to Chloroplast and Eukarya were removed, resulting in 5,195,971 sequences across 18 samples (average of 288,665 sequences per sample). For the ITS dataset, after excluding sequences assigned to Vertebrata and Viridiplantae, 970,898 sequences were retained across 18 samples (average of 53,939 sequences per sample). Sequences that could not be classified at phylum level were also excluded from the analysis, leaving 2,309,591 sequences for 16S rRNA and 528,951 sequences for ITS. The sequence data have been deposited in the NCBI Sequence Read Archive under accession codes (SAMN45951820‐SAMN45951839; SAMN45952464‐SAMN45952483; SAMN45950902‐SAMN45950909; SAMN45951448‐SAMN45951471).

### 2.5 Statistical Analyses

To gain insights into the functional roles of fungi and bacteria, we used ecological guild and functional traits to fungal ASVs based on genus‐level taxonomy derived from ITS rRNA gene data using ‘FUNGuild’ (Nguyen et al. 2016) and ‘FungalTraits,’ (Põlme et al. 2020). Bacterial functional metabolic pathway potential was predicted with ‘PiCrust2’ (q2‐picrust2 2.5.2 plugin) applied to 16S rRNA gene data with QIIME2 (version 2023.2) (Douglas et al. 2019). Metabolic pathways identified by PiCrust2 were manually analysed using the TrypanoCyc database (http://vm‐trypanocyc.toulouse.inra.fr/), which integrates curated pathway information from the MetaCyc database (https://metacyc.org). Pathways were also searched on Google Scholar to assess their relevance to fungi. The metabolic pathways were evaluated for (1) relevance to sulfur cycling, (2) involvement of nicotinamide adenine dinucleotide (NAD), (3) redox‐related functions, (4) roles in fermentation, and (5) potential fungal associations, including complete, incomplete, alternative, or divergent forms. The terms ‘complete’, ‘incomplete’, ‘alternative’, or ‘divergent’ forms relate to whether a pathway has been fully identified in fungi (complete), is partially present or utilised by fungi (incomplete), exists in a different form with the same start and end products (alternative), or if fungi use a distinct pathway to achieve the same metabolic outcome compared to other organisms (divergent). The complete list of pathways is presented in Supporting Information.

Alpha diversity estimators were calculated using the ‘Vegan’ package v2.5–8 (Oksanen et al. 2020) (R‐Core Team, 2022) in the RStudio interface. Differences in alpha diversity were assessed using non‐parametric Kruskal–Wallis tests with treatment factors (time or tissue type), followed by pairwise comparisons using Wilcoxon and Dunn's tests with Bonferroni‐adjusted *p* values. ASV abundances were normalised to relative abundances, and Bray–Curtis dissimilarities were calculated for beta diversity. Principal Coordinate Analysis (PCoA) was performed in Python (OriginPro 2023b, using pandas, NumPy, SciPy, scikit‐learn, seaborn, and Matplotlib) to visualise community composition differences across time, tissue, and their interaction. Community differences were tested using PERMANOVA (999 permutations, pseudo‐F, p), and within‐group variability was assessed using PERMDISP (pseudo‐F, p). Convex hulls were applied to PCoA plots to illustrate group clustering. These analyses were also conducted on microbial class taxa, with key taxa identified from ordination loadings. Group centroids and dispersions were calculated in the ordination space, and the significance of differences in dispersion was tested using ANOVA (Anderson 2006). The difference between ANOVA and PERMANOVA is that ANOVA evaluates differences within‐group dispersion (i.e., variability), while PERMANOVA evaluates differences in average community composition across groups.

For PiCrust2 predicted metabolic pathways, the same non‐parametric tests (Kruskal–Wallis, Wilcoxon, Dunn's) were applied using OriginPro 2023b to test for differences between tissue types. Functional profile shifts across paired samples (e.g., start vs. end, or blade vs. stipe) were further tested with Friedman ANOVA followed by Dunn's post hoc test, corrected by Wilcoxon–Nemenyi–McDonald–Thompson for multiple comparisons. Hierarchical cluster analyses were conducted in OriginPro 2023b using group average clustering with Euclidean distance and/or correlation metrics. The Clustroid Info output was used to identify the most and least representative samples (those closest to the cluster centroid) and the least representative samples (those farthest from the centroid) within each cluster. Results were visualised as dendrograms. For Figures 2, 3, 4, 5, 6, 7, OriginPro 2023b was used, while for Figure 8, Adobe Illustrator 2024 was used.

## 3 Results

### 3.1 Microbial Diversity Across Samples

**FIGURE 1:**
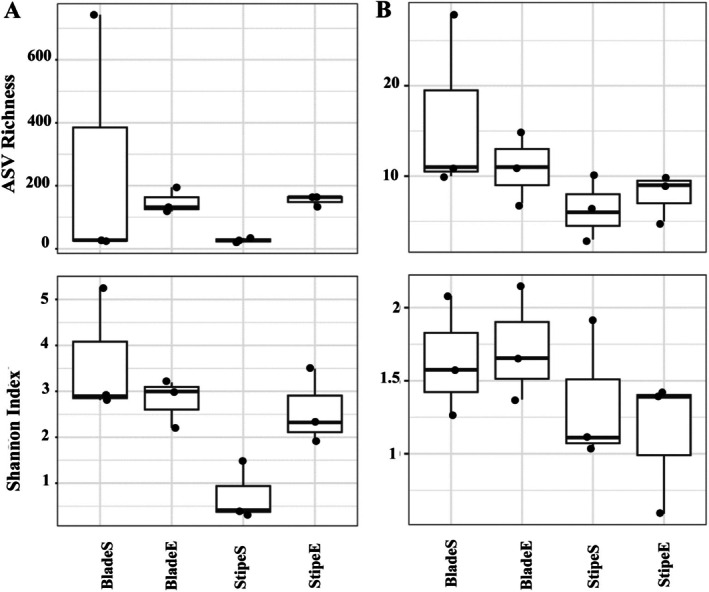
Alpha diversity for bacterial (A) and fungal (B) communities associated with *E. radiata* tissues (“blade” and “stipe”) at day 0 and day 21 of degradation (*n* = 3 per treatment). Diversity was measured using the Shannon index and ASV richness. Error bars indicate standard deviation.

The study analysed 18 kelp samples originating from three *E. radiata* specimens at the beginning and end of a 21‐day degradation experiment in mesocosms and uncovered distinct patterns in the diversity and composition of bacterial and fungal communities. The fungal community comprised 424 ASV, and the fungal sequences were assigned to 5 phyla and 20 classes, exhibiting lower diversity than bacteria (Figure 1).

**FIGURE 2:**
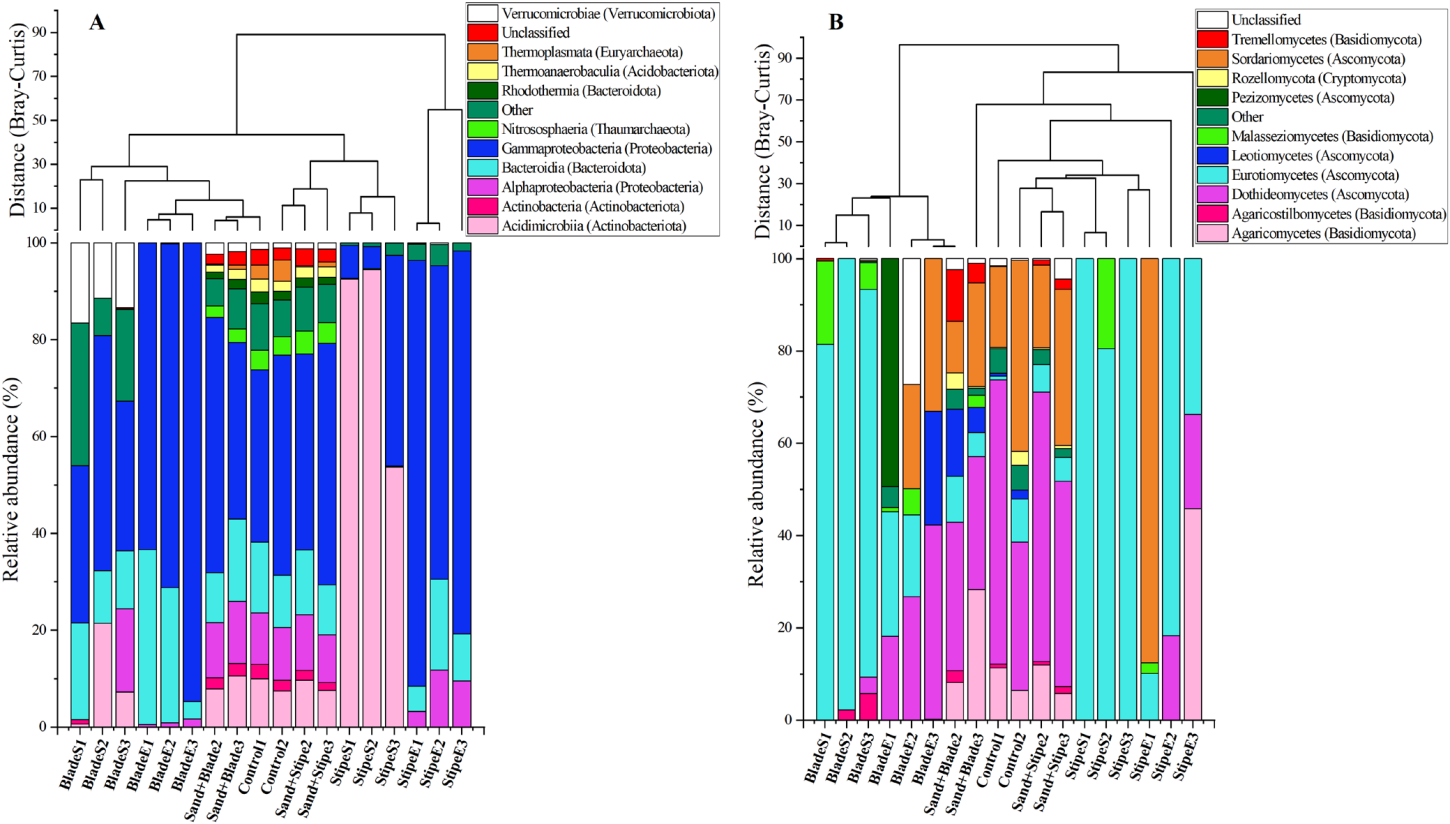
Community composition based on relative abundance at days 0 and 21 at class level (corresponding phylum is in parentheses), combined with hierarchical cluster analysis (sum of distances) for (A) “bacterial” (B) “fungal” community during *E. radiata* degradation. “Other” refers to microbial classes that were classified but did not belong to the dominant classes shown, while “unclassified” includes taxa identified only to the phylum level, with no class‐level assignment. The dendrogram shows hierarchical clustering of microbial communities based on relative abundance profiles, using group average linkage and Bray–Curtis dissimilarity. The clustering reflects similarity in community composition across sample types. Among bacterial communities, the “sand + stipe” sample showed the highest similarity to all other samples and was the most representative sample in the hierarchical clustering, while the stipe start was the least representative. For fungal communities, the “sand + blade” sample was the most representative, whereas the “stipe end” sample was the least representative.

Ascomycota dominated all samples (83.7% of relative sequence abundance and 75% of total ASV count), while Basidiomycota represented a smaller portion (15.3% of relative abundance and 20% of total ASV count). The major fungal classes observed throughout the kelp degradation were Eurotiomycetes, Dothideomycetes, Sordariomycetes, and Agaricomycetes, with temporal variations in their abundance throughout the study (Figure 2). Within Eurotiomycetes, the most abundant genus was *Aspergillus*, while *Stemphylium* and *Fusarium* were the most abundant genera within Dothideomycetes and Sordariomycetes, respectively.

The bacterial community encompassed 10,976 ASVs across 45 phyla and 110 classes (Figure 1). The phylum Proteobacteria was predominant, constituting 63% of all sequences and 41% of ASVs, prominently featuring Bacteroidota and Actinobacteria. Notable classes included Gammaproteobacteria, Bacteroidia, Alphaproteobacteria, and Acidimicrobiia (Figure 2). Alpha diversity (Shannon index and richness) was compared between time points (day 0 vs. day 21) and tissue types (blade, stipe) for both bacterial and fungal communities. All groups exhibited relatively high diversity, with slight increases in richness and Shannon index values after 21 days of degradation (Figure 1).

### 3.2 Community Dynamics Over Time

**FIGURE 3:**
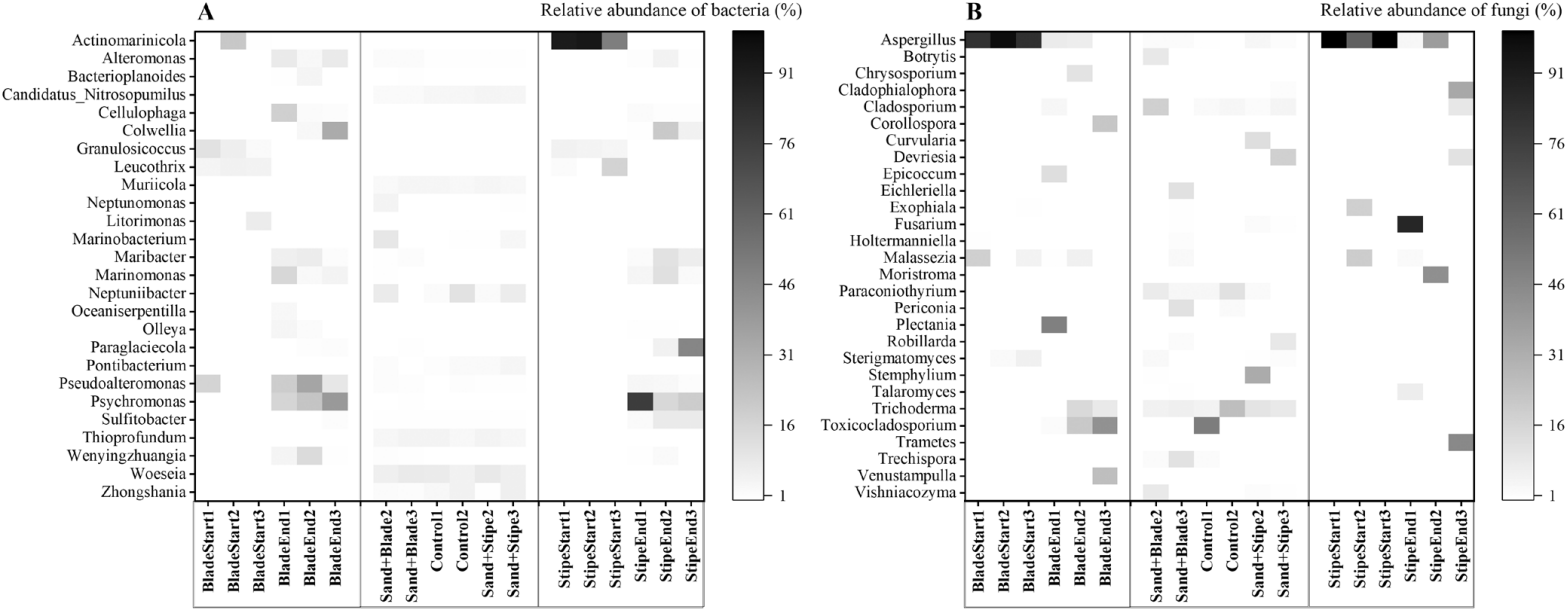
Heatmap of the most abundant “bacterial” (A) and “fungal” (B) genera based on ASV data from the 16S rRNA and ITS rRNA gene sequences during *E. radiata* degradation. ASVs were aggregated at the genus level, and only the most frequent genera (by mean relative abundance across all samples) are shown. The *x*‐axis represents the 18 samples across different tissue types and time points; while the *y*‐axis lists the dominant genera. Cell colour intensity reflects the relative abundance (%) of each genus after normalisation.

A heatmap detailing temporal shifts in relative abundances of key bacterial and fungal genera (Figure 3) showed that the bacteria *Psychromonas* and *Pseudoalteromonas* increased in abundance over time, while *Actinomarinicola* declined; among fungal genera, *Aspergillus* decreased, whereas *Toxicocladosporium*, *Trametes*, *Fusarium*, and *Cladophialophora* demonstrated strong growth by day 21.

**FIGURE 4:**
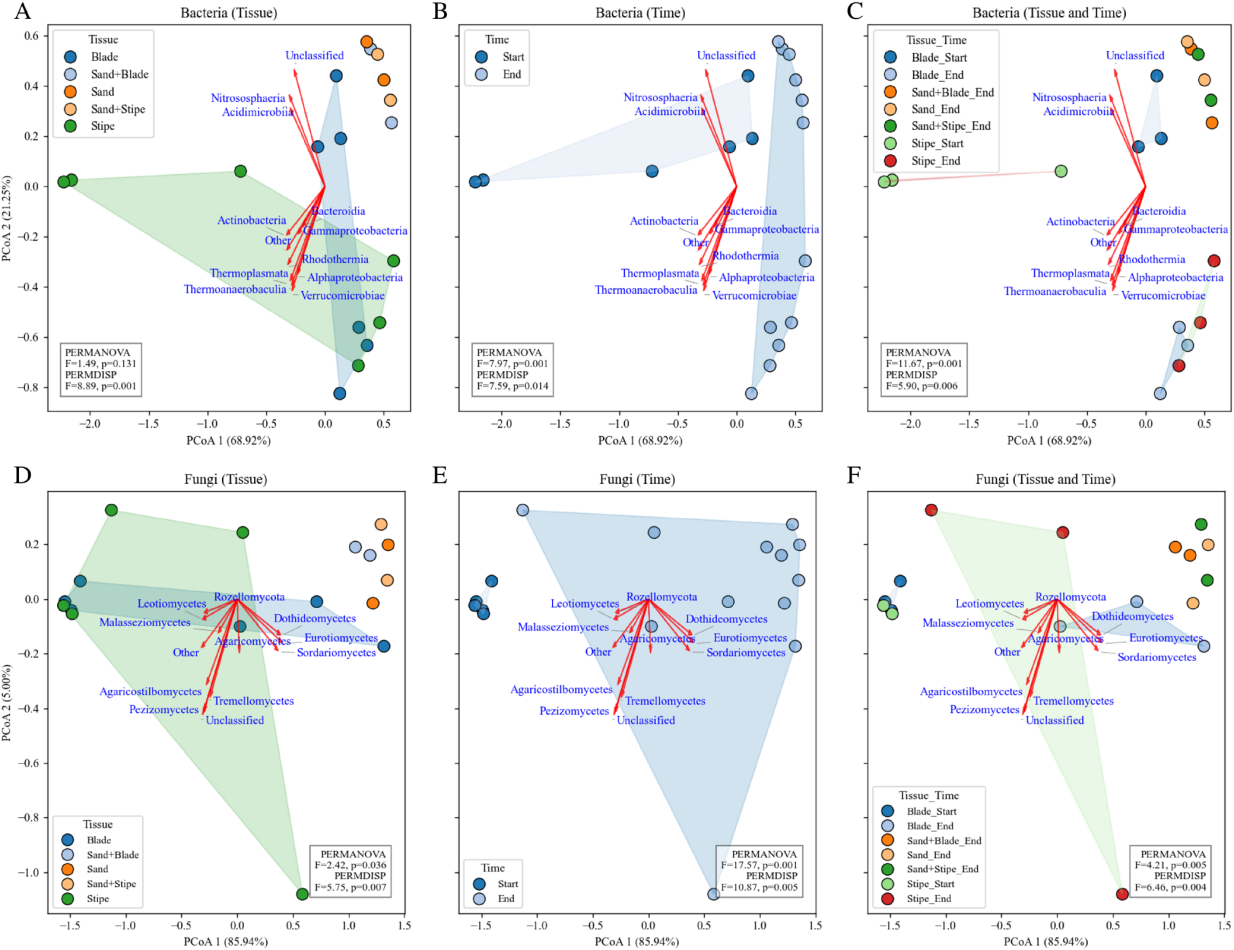
Principal coordinate analysis (PCoA) comparing bacterial (A–C) and fungal (D–F) community composition during *E. radiata* degradation, showing variation across tissues, sampling times, and their interactions. Ellipses highlight significant grouping patterns, and red vectors represent key microbial classes responsible for community dissimilarity. Axis labels show the percentage of variation explained by each coordinate. PERMANOVA results (assessing community differences) and PERMDISP results (testing dispersion homogeneity) are shown in each plot, with detailed statistics in Tables S2 and S3. “Other” refers to classified but non‐dominant classes; “unclassified” includes taxa resolved only to the phylum level.

The PCoA revealed distinct structuring of bacterial and fungal communities and ASVs during *E. radiata* degradation across tissue types and sampling times. For microbial communities (Figure 4A–F showing taxonomic classes), PCoA 1 explained most of the variation, accounting for 68.9% in bacteria and 85.9% in fungi, while at the ASV level (Figure S2A–F), PCoA 1 explained 49.4% and 30.9% of bacterial and fungal variation, respectively. At taxonomic class level, PERMANOVA showed significant effects of time (bacteria: *F* = 7.97, *p* = 0.001; fungi: *F* = 17.57, *p* = 0.001) and tissue × time interactions (bacteria: *F* = 11.67, *p* = 0.001; fungi: *F* = 4.27, *p* = 0.004), with tissue effects significant only for fungi (*F* = 2.42, *p* = 0.022) but not bacteria (*F* = 1.49, *p* = 0.156). At the ASV level, tissue, time, and interactions were all significant for both bacteria (*F* = 1.66–3.63, *p* ≤ 0.008) and fungi (*F* = 1.32–3.55, *p* ≤ 0.020). PERMDISP analyses revealed significant within‐group variability at both class and ASV levels (bacteria: *F* = 8.98, *p* < 0.001; fungi: *F* = 7.59, *p* = 0.014; bacterial ASVs: *F* = 67.93, *p* = 0.000; fungal ASVs: *F* = 10.12, *p* < 0.001), while posthoc PERMANOVA confirmed highly significant temporal shifts, particularly in fungal classes and ASVs (*p* < 1 × 10^−14^) (Tables S2 and S3). Ordination loadings in the PCoA plots highlighted taxa contributing most strongly to the observed patterns, suggesting that groups such as Gammaproteobacteria, Bacteroidia, Alphaproteobacteria, and Actinobacteriota aligned with bacterial variation, while Tremellomycetes, Agaricomycetes, Rozellomycota, Malasseziomycetes, and Eurotiomycetes were prominent in fungal ordination patterns (Figure 4A–F). These patterns are exploratory visual indications from ordination and are not a test of differential abundance.

### 3.3 FUNGuild and FungalTraits to Determine Ecological Roles of Fungi

**FIGURE 5:**
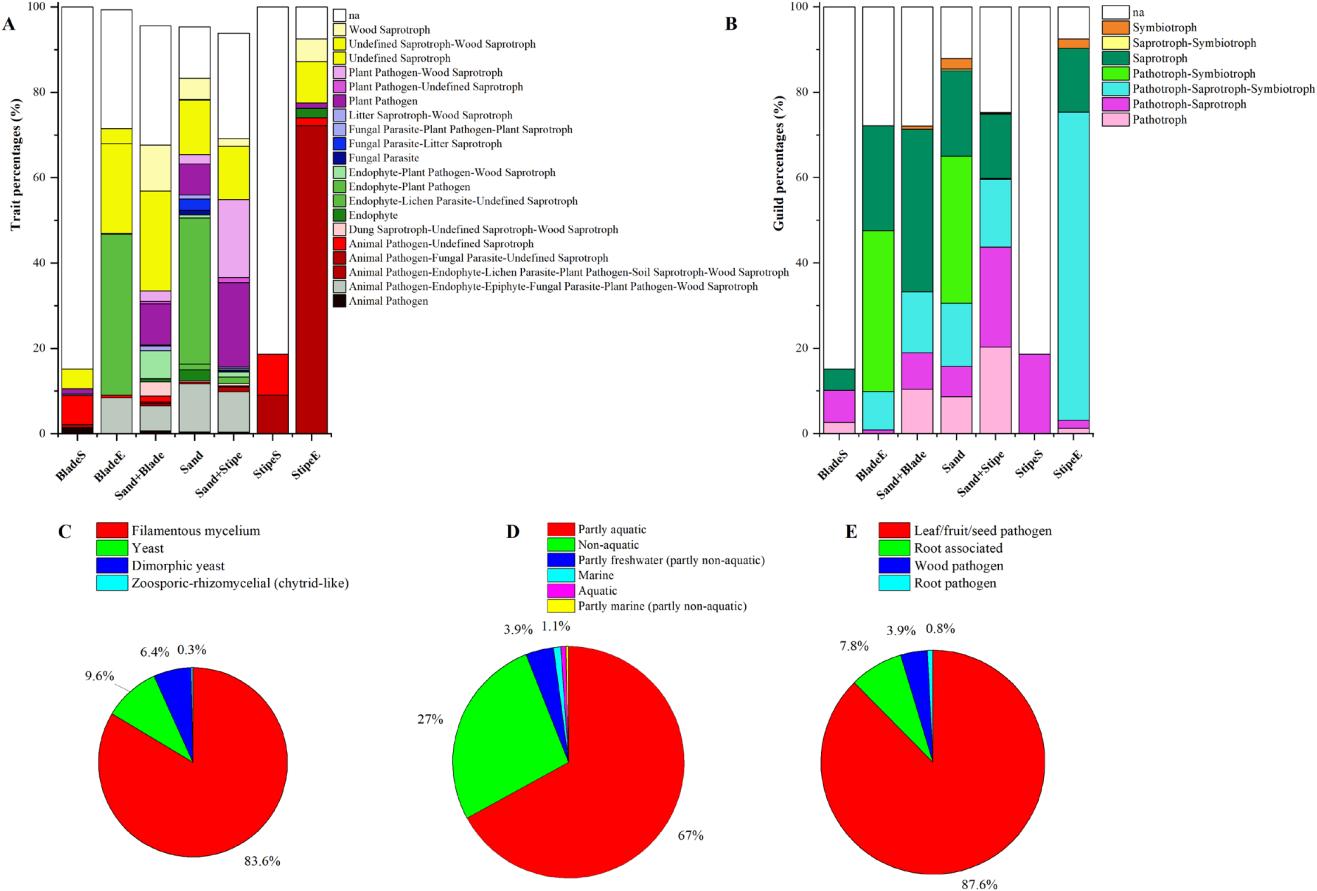
Ecological traits and guild assignments of fungal ASVs during *Ecklonia radiata* degradation, based on FUNGuild and FungalTraits databases, with proportions calculated from the relative abundance of annotated ASVs. (A) Partial list of secondary lifestyle classification from the traits template in FUNGuild; categories representing less than 1% are shown in Figure S3. (B) Ecological guild classification based on the trophic mode template from FUNGuild. (C) Fungal body type classification from FungalTraits. (D) Biotrophic mode classification from FungalTraits. (E) Decay type and pathogenic capacity classification from FungalTraits. “na” indicates ASVs that could not be assigned to any category.

A substantial portion of the fungal community, especially at the onset of degradation, could not be assigned to the phylum level (43% of ASVs, 39% of sequences), compared to 9% of ASVs and 56% of sequences for bacteria. This taxonomic limitation extended to functional annotations, with 27%–74% of fungal ASVs unclassified (‘na’) across FUNGuild and FungalTraits templates (Table 1, Figure 5A–E). Despite these limitations, the fungal community during *E. radiata* degradation was functionally diverse. Saprotrophs became more prominent by day 21, especially in the fungal community associated with sand and kelp samples (Sand + Blade and Sand + Stipe). While sand‐associated samples supported greater trait diversity, including parasitic and endophytic fungi. Guild classifications revealed increasing dominance of mixed strategies (e.g., pathotroph–saprotroph) in later stages. Overall, most fungi were filamentous (83.6%), partly aquatic (67%), and frequently annotated as leaf/fruit/seed pathogens (87.6%).

**TABLE 1:** The seven most frequently occurring fungal ecological categories across FungalTraits (FT) and FUNGuild (FG) databases.

Database	Template	1	2	3	4	5	6	7
FT	Secondary lifestyle	Saprotroph (wood, soil, litter, undefined, pollen, dung, nectar/tap)	N/A	Plant pathogen	Parasite (myco, animal)	Foliar endophytes	Epiphyte	Sooty mould
	%	43.50	27.42	16.78	9.69	1.89	0.47	0.24
FT	Body type	Filamentous mycelium	NA	Yeast	Dimorphic yeast	Zoosporic rhizomycelial (chytrid‐like)		
	%	61.47	26.71	7.09	4.73	0.24		
FT	Trophic type	Partly‐aquatic	NA	Non‐aquatic	Partly freshwater (partly non‐aquatic)	Marine	Aquatic	Partly marine (partly non‐aquatic)
	%	45.15	32.86	18.2	2.6	0.71	0.47	0.24
FT	Comment lifestyle	N/A	Endophyte (foliar, root, dark septate)	Saprotroph (unspecified, wood, nectar, litter)	Plant pathogen	Decomposer (fungal, animal)	Root‐associated	Rock‐inhabiting
	%	54.14	19.62	15.37	5.44	3.31	1.89	0.24
FT	Pathogenic capacity	Foliar endophytes	NA	No endophytic capacity	Root associated	Root endophyte dark septate		
	%	59.57	28.61	8.75	1.89	1.42		
FT	Endophytic interaction	N/A	Hypervariable	Toxin producing parasite (animal, myco, some species)	Extremophile	Litter saprotroph_some	Mycoparasite	Pathogen (plant, wood)
	%	74	17.73	2.6	1.65	0.95	2.36	0.47
FG	Guild	N/A	Saprotroph	Pathotroph‐Saprotroph‐Symbiotroph	Pathotroph‐Saprotroph	Pathotroph	Pathotroph‐Symbiotroph	Symbiotroph
	%	38.3	24.35	12.53	10.87	10.17	1.89	1.42
FG	Confidence ranking	N/A	Microfungus	Facultative Yeast‐Microfungus	Corticioid	Yeast	Agaricoid	Polyporoid
	%	63.36	24.82	4.02	2.6	1.65	1.42	0.95

*Note:* Each row presents the percentage of ASVs assigned to each trait category within a given template (e.g., lifestyle, body type, trophic type). Percentages reflect the proportion of annotated ASVs in each dataset. Categories were ranked based on their abundance. In the FG database, “Guild” refers to ecological trophic modes such as saprotroph, symbiotroph, or pathotroph.

### 3.4 PiCrust2 Predicted Metabolic Pathways

**FIGURE 6:**
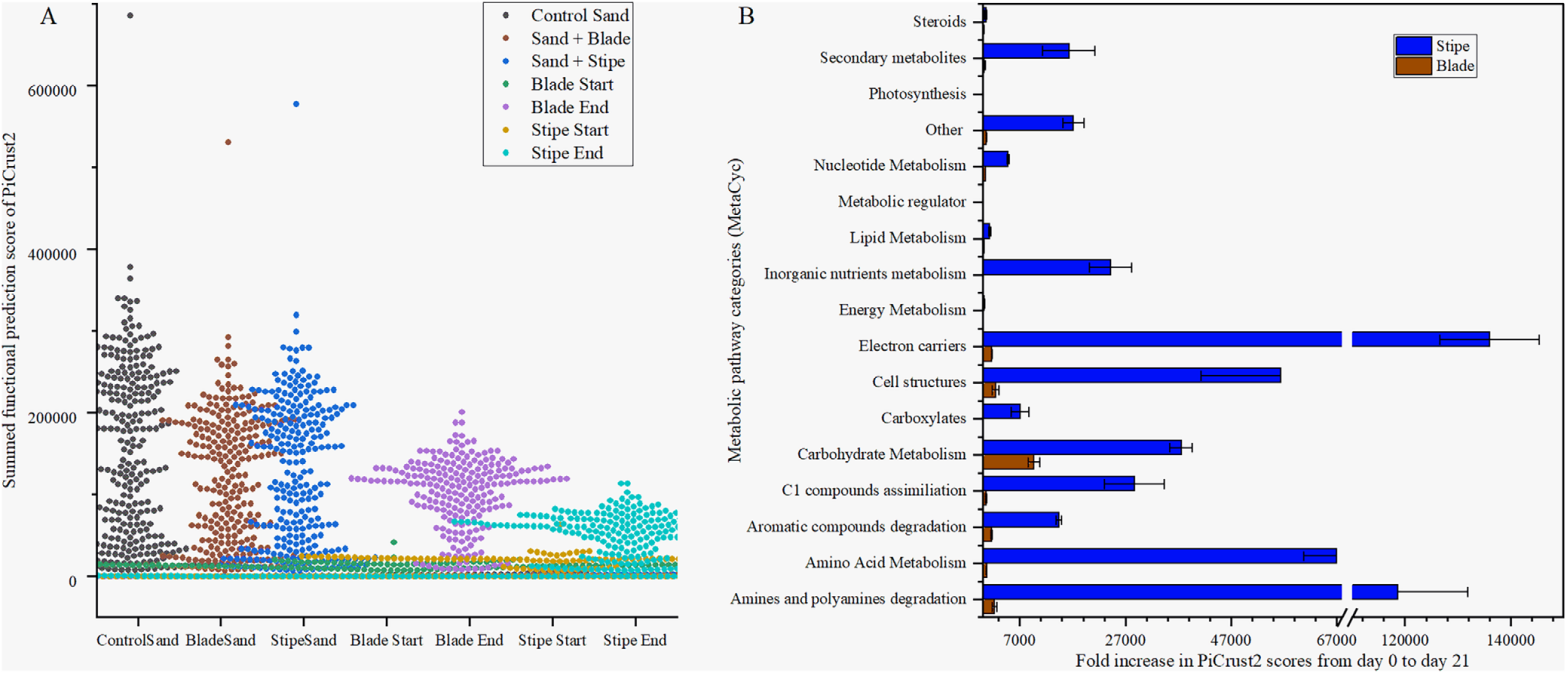
Predicted functional profiles of metabolic pathways from PiCrust2 during *E. radiata* degradation from day 0 to day 21. (A) Beeswarm plot showing the overall distribution of raw PiCrust2 pathway scores for each sample group. Sand exhibited the highest baseline functional potential with more variability, while tissue samples (blade and stipe) show lower baseline values but more pronounced increases over time. (B) Bar chart showing the relative abundance of predicted metabolic pathway categories (based on MetaCyc classification) in blade and stipe tissues from start to end of degradation (21 days). Error bars represent the standard deviation among replicates. Figure S4 provides a detailed breakdown of pathway categories and superclasses associated with each tissue type (blade and stipe).

PiCrust2 analysis revealed significant metabolic differences in predicted pathways across time points and tissue types (Figure 6A, Kruskal‐Wallis, Friedman ANOVA *p* < 0.0001 Table S5), with all pairwise comparisons significant, except between blade and stipe in the sand (*p* = 0.11391, Table S6).

**FIGURE 7:**
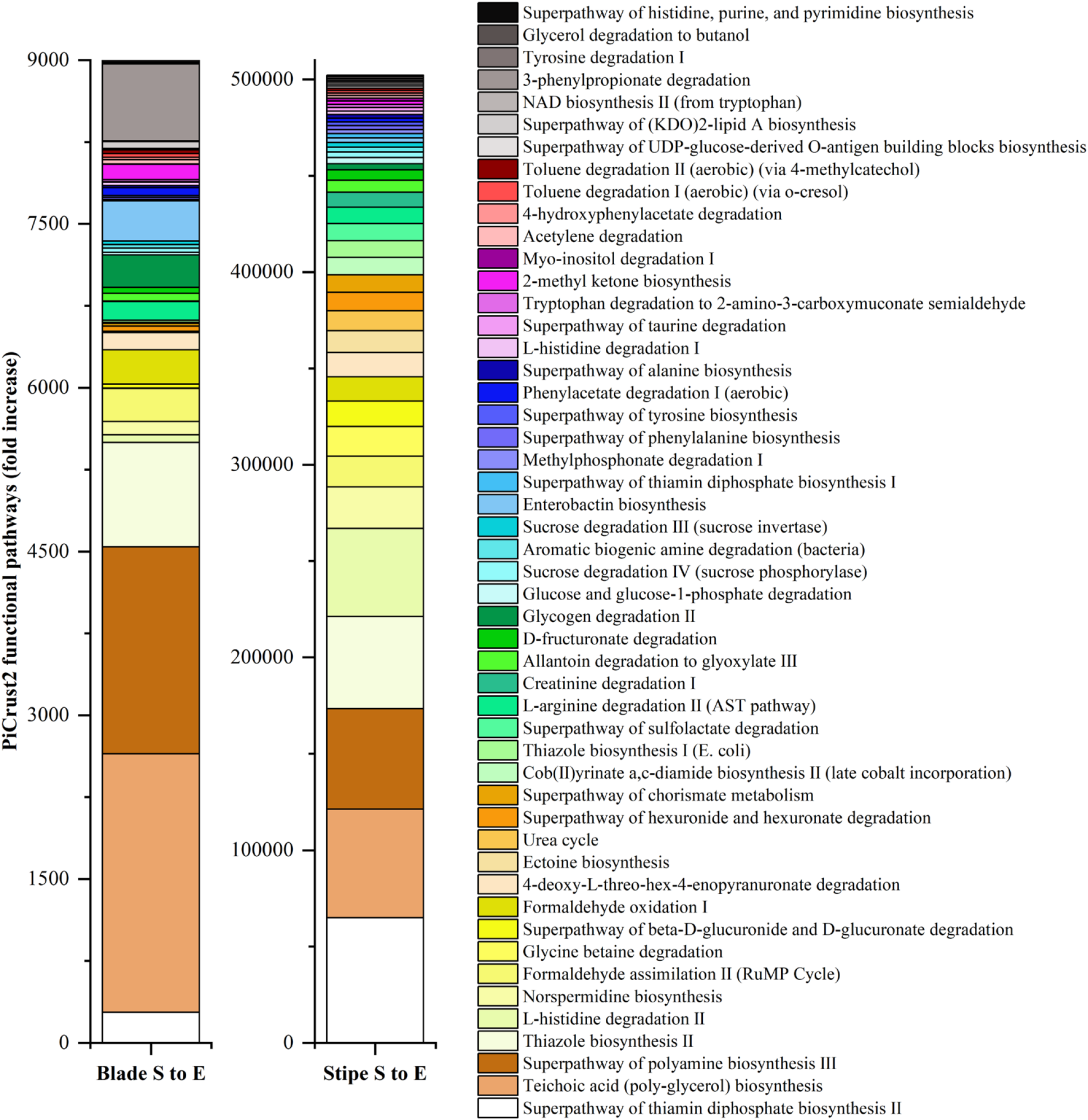
Stacked bar plot illustrating the top 50 metabolic pathways predicted by PiCrust2 that showed the greatest fold increase from day 0 to day 21 during *E. radiata* degradation. Each bar represents the average of the three replicates per each tissue type (blade and stipe), *n* = 3. Pathway abundances were averaged to emphasise broader trends in metabolic functional potential in the different tissue types. These metabolic predictions are based on 16S rRNA gene data.

Overall, important metabolic pathways in the blade included carbohydrate metabolism, cell structure, amines and polyamines, electron carriers, and aromatic compounds (Figure 6B). In contrast, important metabolic pathways in the stipe were electron carriers, amines and polyamines, amino acid metabolism, cell structure, carbohydrate metabolism, C1 compounds, inorganic nutrient metabolism, and secondary metabolites. Hierarchical clustering showed that the “sand + blade” combination was the most representative treatment, and NAD^+^ synthesis was the most representative pathway (data not shown). Although both tissues shared several key metabolic pathways (Figure 6B and Figure 7): teichoic acid biosynthesis (TEICHOICACID‐PWY, blade 2367‐fold, stipe 56314‐fold), polyamines biosynthesis (PWY‐6565, blade 1897‐fold, stipe 52198‐fold), glucose and glucose‐1‐phosphate degradation (GLUCOSE1PMETAB‐PWY, blade 24‐fold, stipe 3139‐fold), CMP‐legionaminate biosynthesis (PWY‐6749, blade 8900‐fold, stipe 508‐fold), catechol degradation to β‐ketoadipate (CATECHOL‐ORTHO‐CLEAVAGE‐PWY, blade 63‐fold, stipe 786‐fold), histidine degradation (HISDEG‐PWY, blade 32‐fold, stipe 1804‐fold), superpathway of hexuronide and hexuronate (GALACT‐GLUCUROCAT‐PWY, blade 48.07, stipe 9426.68), superpathway of histidine, purine and pyrimidine biosynthesis (PRPP‐PWY, Blade 32.11, Stipe 870.14) (Figure 7, Table S1).

The metabolic pathway analysis of PiCrust2 revealed that eight pathways are documented on the MetaCyc database as chimeric (requiring contributions from multiple organisms), and 81 of the 423 pathways are related solely to bacteria or have not been studied in fungi (Table S1, Figures S5–S7). Overall, 342 of all 423 metabolic pathways have been documented for fungi (MetaCyc database and on Google Scholar) (either pathways exist, in divergent, alternative, or incomplete forms), including 65 pathways on the MetaCyc database, of which eight pathways are chimeric. Moreover, of the 423 pathways, 247 are involved in sulfur cycling—138 directly and 109 indirectly—either by containing sulfur atoms/compounds or through coenzyme A, with 194 of these documented for fungi (39 on the MetaCyc database of which six are chimeric pathways). Furthermore, 259 are NAD‐related, with 220 documented for fungi, including 37 on the MetaCyc database, of which seven are chimeric. Additionally, 320 pathways are redox‐related (242 directly and 78 indirectly), and among these, 281 are documented for fungi, including 47 on the MetaCyc database, of which five are chimeric pathways.

## 4 Discussion

In this study, we investigated the degradation of *E. radiata* kelp tissues (blade and stipe) over 21 days under simulated beach conditions in a mesocosm setup, focusing on changes in bacterial and fungal communities and their functional roles. We aimed to expand our prior findings that fungi play a key role in kelp remineralisation and drive inorganic carbon (DIC, TA) and DMSP production during *E. radiata* remineralisation (Perkins et al. 2023). Given the lack of equivalent tools like PiCrust2 to predict functional metabolic potential for fungi from the sequencing results, we used the bacterial metabolic pathways to (1) identify any links to sulfur processing and (2) uncover any documented fungal pathways, using the MetaCyc database and Google Scholar search. Our analysis revealed a diverse bacterial and fungal communities involved in the remineralisation of *E. radiata*. Of the 423 identified bacterial pathways, 342 have also been documented for fungi in the literature, either in complete, divergent, alternative, or incomplete forms. Notably, 247 metabolic pathways directly or indirectly contribute to sulfur cycling, of which 194 are also documented for fungi.

Blade and stipe shared many pathways, but notable differences were observed in the increases and range of metabolic pathways of the stipe (Figures 6 and 7, Table S1). Carbohydrate metabolism dominated blade degradation (e.g., CMP‐legionaminate biosynthesis I and glycogen degradation II), whereas electron transport processes (e.g., thiamine diphosphate biosynthesis I and II, thiazole biosynthesis I and II, cob (II)yrinate a,c‐diamide biosynthesis II, and NAD biosynthesis and salvage pathways), along with amine and polyamine degradation pathways (e.g., polyamine biosynthesis III, norspermidine biosynthesis, glycine betaine degradation, ectoine biosynthesis, creatinine degradation I, allantoin degradation to glyoxylate III, and aromatic biogenic amine degradation), were more prominent in the stipe.

Electron transport and amine and polyamine degradation dominated the stipe (Figure 6B), reflecting structural breakdown and demanding higher energy via oxidative phosphorylation. This metabolic environment supports a specialised microbial community, with the stipe emerging as a prime niche for fungi (Figures 2, 4, and 5). By day 21, fungal reads increased (27,215–71,918), while bacterial reads declined (1,217,503–860,011; Table S4), though these trends must be interpreted cautiously due to amplicon sequencing biases. Distinct fungal clustering (PERMANOVA, *F* = 2.42, *p* = 0.022; PERMDISP, *F* = 7.59, *p* = 0.014; Figure 4D), spatial heterogeneity, and strong tissue × time interactions (PERMANOVA, *F* = 17.57, *p* = 0.001; *F* = 4.27, *p* = 0.004) point to high fungal flexibility and niche specialisation. These patterns align with prior work on fungal metabolic plasticity (Alster et al. 2021; Wrzosek et al. 2017) and their disproportionately important role in kelp degradation (Perkins et al. 2023), consistent with terrestrial systems where fungi persist or increase as bacterial abundance remains stable or declines (Dong et al. 2021; Wang et al. 2022). In contrast, bacterial composition was shaped by temporal shifts (PERMANOVA, *F* = 7.97, *p* = 0.001) and significant tissue × time interactions (*F* = 11.67, *p* = 0.001; Figure 4A–C), although these significant effects may reflect the reduction observed in raw reads. Bacterial communities were more compositionally constrained than fungal communities; ASV‐level analyses revealed substantial within‐group variability. These results provide a basis to explore how bacterial and fungal communities shift over time during kelp degradation, alongside the metabolic pathways involved.

### 4.1 Bacterial Community Diversity

The bacterial community on day 0 (endophytic) was more diverse, particularly in the blade, compared to the community on the degrading kelp by day 21 (Figure 2A). Acidimicrobiia were abundant in the stipe on day 0, suggesting a potential association with the stipe's structural complexity or chemical composition. In contrast, Gammaproteobacteria and Bacteroidia dominated the bacterial community in most samples, reflecting their documented roles in other kelp studies (Brunet et al. 2021; Bacci et al. 2015; Phelps et al. 2021; King et al. 2023) and marine organic matter degradation (Dyksma et al. 2016; Gutierrez 2017). While not all members of these classes may be functionally involved in degradation, many genera within them—such as *Psychromonas* and *Pseudoalteromonas* (Gobet et al. 2018; Xu et al. 2021)—have been previously associated with algal polysaccharide degradation. These two classes are well‐recognised for their roles in degrading complex carbohydrates and proteins. Moreover, Alphaproteobacteria, including *Sphingomonadales* (found exclusively in sand samples with or without kelp), are known for hydrocarbon degradation (Gutierrez 2017), reflecting niche differentiation based on substrate specialisation. For both fungi and bacteria, sand‐associated environments supported greater diversity (Figures 2 and 3), likely due to mixed organic matter inputs from terrestrial and marine sources (Stal 2016).

### 4.2 Fungal Community Diversity

On day 0, Eurotiomycetes, particularly *Aspergillus*, *Penicillium*, and to a lesser extent, *Talaromyces*, dominated the fungal community in both tissues, suggesting an endophytic origin. Yet, these fungi were largely categorised as “unclassified” in FUNGuild and FungalTraits (Figure 5A,B). By day 21, Dothideomycetes, Sordariomycetes, Leotiomycetes, and Pezizomycetes became more prominent, reflecting a shift in community composition toward saprotrophic origin. Dothideomycetes and Sordariomycetes have been associated with other kelp species (Perkins et al. 2021; Flewelling et al. 2013; Rashmi 2019), different hosts (Purahong and Krüger 2012), and the degradation of recalcitrant organic matter (e.g., cellulose (Jones et al. 2019; Samaradiwakara et al. 2023; Schneider et al. 2012), or a structurally embedded protein (Baltar et al. 2021; Breyer et al. 2022)) in marine and terrestrial environments (Tedersoo et al. 2014). This shift in community composition was further supported by secondary lifestyle and ecological guild classification (Figure 5A,B), as well as by the clear temporal separation in sample dispersion (PERMDISP: *F* = 10.87, *p* = 0.005; Figure 4B), consistent with increased heterogeneity in the fungal community by day 21. The FungalTraits identified 67% of fungi as partly aquatic, suggesting a broad ecological niche, with only 1% identified as of marine origin (Figure 5D). Despite the evident role of fungi in organic matter degradation, functional characterisation remains limited, reflecting the dominance of terrestrial‐derived data and the constraint of current databases.

Various other databases support fungal taxonomy and functional annotation, including UNITE (https://unite.ut.ee/repository.php), MycoBank (https://www.mycobank.org/), and Index Fungorum (https://indexfungorum.org/). Yeast‐specific resources include the Saccharomyces Genome Database (https://www.yeastgenome.org/) and Candida Genome Database (http://www.candidagenome.org/), which provide detailed genomic and functional data. Strain collections such as American Type Culture Collection (https://www.atcc.org/microbe‐products/mycology/yeast#t=productTab&numberOfResults=24 and https://www.atcc.org/microbe‐products/mycology#t=productTab&numberOfResults=24), the Yeast Strain Collection (https://www.ncyc.co.uk/), the Phaff Yeast Culture Collection (https://phaffcollection.ucdavis.edu/), and Horizon's Yeast Knockout Collection (https://horizondiscovery.com/en/non‐mammalian‐research‐tools/products/yeast‐knockout) support experimental research. Emerging tools like EsMeCaTa (Belcour et al. 2022) and FunOMIC (Xie and Manichanh 2022) show promise for functional prediction in fungal communities; however, they are not yet as well‐established as PiCrust2 and are also limited in their applicability for environmental fungi.

### 4.3 Functional Roles and Sulfur Processing Pathways Based on PiCrust2

Since PiCrust2 was used to predict bacterial metabolic pathways, we extended the analysis to identify links to sulfur processing and manually searched databases to determine whether fungi have been documented in these pathways. Although this unconventional approach may invite scepticism, as PiCrust2 is not specifically designed for fungi, it provided a broader perspective on ecological roles and potential contributions (Figures S5–S7).

Many bacteria in this study are documented for sulfur cycling, including Proteobacteria (Qian et al. 2024) (e.g., *Sulfitobacter* (Deng et al. 2024), *Desulfatiglans* (Wasmund et al. 2017), *Neptunomanas, Thiothrix, Thioclava, Thiohalophilus, Thiogranum, Thiorhodospira, Hydrogenovibrio, Sulfurifustis, Thiohalorhabdales, Leucothrix, TX1A‐55*), Actinobacteria (Qian et al. 2024), *Desulfitobacterium* (Dyksma et al. 2018; Nie et al. 2021) and Desulfobacteria (Dyksma et al. 2018; Nie et al. 2021). Among fungi, *Aspergillus* (Traynor et al. 2019; Amich et al. 2016), *Fusarium* (Abe et al. 2007; Bacic and Yoch 1998; Xu et al. 2018), *Penicillium* (Fan et al. 2022; Chen et al. 2021; Pócsi et al. 2004), and *Trametes* (Gao et al. 2017) have also been documented for sulfur oxidation, sulfate reduction, or transformation of sulfur compounds (Fan et al. 2022). However, sulfur cycling of fungi, especially concerning organosulfur compounds (e.g., sulfides, sulfones, sulfonates, sulfate esters, and sulfamates), remains underexplored (Linder 2012, 2018). Nonetheless, fungi may contribute to sulfur cycling through other means (Gahan and Schmalenberger 2014; Marzluf 1993, 1997; Allen and Shachar‐Hill 2009).

One mechanism by which fungi can drive bacterial sulfur cycling is by creating a nitrogen‐limited environment during kelp degradation, which indirectly shifts the bacterial community to rely on alternative electron‐accepting pathways, such as sulfur cycling (Kopáček et al. 2013). This can enhance the presence of sulfur‐cycling bacteria. Moreover, amine and polyamine degradation were prominent during kelp degradation in both kelp tissues (blade and stipe, Figure 6), and fungi are efficient in oxidising amines (Stenholm et al. 2021; Yamada et al. 1965; Yagodina et al. 2002). Oxidative deamination releases ammonia and aldehydes, while transamination transfers amino groups (Stenholm et al. 2021; Brunke et al. 2014; Stewart et al. 2018). These nitrogen‐limiting processes can stimulate sulfur‐cycling microbes by driving the production of sulfur reductase and sulfite oxidase (Tian et al. 2017), while increasing redox conditions that are naturally present during kelp degradation. Fungal peroxidase production as a byproduct of oxidative metabolism (Bhat and Gogate 2021) may further influence these processes indirectly by increasing redox potential.

Metabolic processes during *E. radiata* degradation (e.g., carbohydrate metabolism and amine degradation (Figure 6B)) rely heavily on redox reactions, particularly the recycling of NAD^+^ (Teodoro et al. 2013). NAD^+^ assists in ATP production (Luengo et al. 2021), biosynthesis (Yaku et al. 2019), DNA repair (Amjad et al. 2021), endosymbiotic gene transfer (Ternes and Schönknecht 2014), pathogenesis (Roussin and Salcedo 2021), stress responses, signalling pathways (Amjad et al. 2021), secretory processes (Fischer and Glass 2019), pathogenicity (Strømland et al. 2021) and functional divergence (Marcet‐Houben et al. 2009). NAD^+^ was the most representative pathway to *E. radiata* remineralisation and the recycling of NAD^+^ was observed in 259 of 423 pathways, of which 220 are documented in fungi. NAD^+^ showed a significant 990‐fold increase in the stipe and a 5.7‐fold increase in the blade after 21 days of degradation (Table S1). NAD^+^ enables fungi to adapt to low oxygen conditions (Shimizu 2018), likely also during *E. radiata* degradation. Low oxygen conditions during kelp degradation constrain respiration, making NAD^+^ regeneration crucial for microbial activity. NAD^+^ drives energy production through aerobic glycolysis (Bokor et al. 2022), the TCA cycle, and oxidative phosphorylation (Marcet‐Houben et al. 2009; Boyer et al. 1977; de Assis et al. 2021), while intermediates such as glucose, pyruvate, and acetyl‐CoA can be directed into multiple fermentation pathways (Bai et al. 2017; Studer et al. 2016; Cameron et al. 2012; Kraan 2013), supporting ATP production and redox balance. Fungi can facilitate redox cycling during mineralisation through peroxidase production (Krueger et al. 2016; Yu et al. 2019) and NAD^+^ release (Asada et al. 1986; Kido et al. 2015).

**FIGURE 8:**
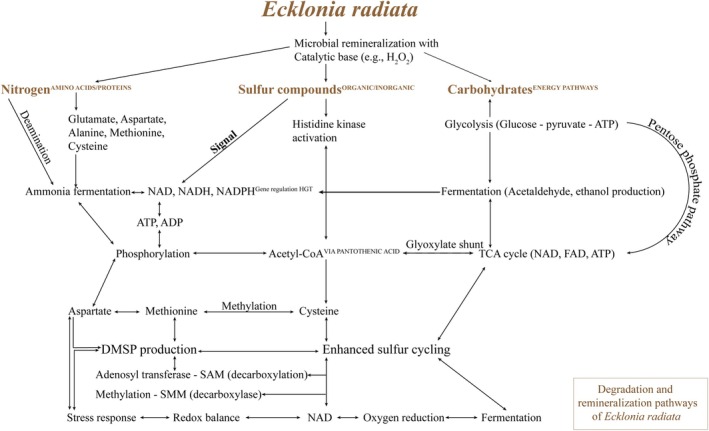
Schematic overview of microbial degradation and remineralization pathways during *E. radiata* degradation. This diagram illustrates microbial contributions to nitrogen, sulfur, and carbon cycling, integrating predicted bacterial metabolic functions (from PICRUSt2) and fungal contributions supported by the documentation from the MetaCyc database and literature (Table S1). Key metabolic processes represented during *E. radiata* degradation include glycolysis, fermentation, the TCA cycle, and sulfur compound transformations, amine and polyamine degradation, ammonia fermentation, phosphorylation, DMSP‐related sulfur cycling, and redox cofactor (NAD^+^, NADH, NADPH) processes. While all pathways may involve fungal activity, redox and NAD‐linked processes are likely prominently driven by fungi and may initiate broader microbial interactions. These processes facilitate energy transfer and regulatory signalling between metabolic pathways. Figures S5–S7 were generated based on fungal metabolic pathways collated in Table S1, grouped by superclasses and categories, and emphasising those related to sulfur cycling that may be linked to DMSP production.

Another mechanism by which organisms drive organic matter remineralisation is to utilise foundational pathways such as NAD^+^ and redox, or to produce toxic compounds. These activities impose selective pressure that shapes the microbial community composition (Singh et al. 2013). Yeasts and fungi regenerate NAD^+^ during ethanol production through alcoholic fermentation to conserve energy (Jeffries 1983). Additionally, amine oxidases catalyse the conversion of amines into toxic aldehydes, ammonia, and hydrogen peroxide. A simplified diagram (Figure 8) based on PiCrust2 results illustrates the potential connection between these metabolic pathways and sulphur cycling during kelp degradation. The diagram emphasises the roles of acetaldehyde and acetyl‐CoA in ethanol production and sulphur cycling, leading to methionine metabolism. Methionine, derived from these pathways, serves as a precursor to DMSP. Methionine adenosyltransferase converts methionine into S‐adenosylmethionine (SAM), which is then decarboxylated and methylated to produce DMSP by DMSP synthase. Alternatively, methionine can be converted into S‐methylmethionine (SMM) by methionine S‐methyltransferase (MmtN), which is then transformed into DMSP in an NAD (P)H‐dependent reaction.

### 4.4 Final Thoughts for Fungal Challenges

Our goal was not to make definitive claims about fungal metabolism using PiCrust2, but rather to emphasise the numerous pathways through which fungi may contribute to sulfur cycling during *E. radiata* degradation, while also noting that the current lack of tools continues to limit fungal research. PiCrust2, though unconventional for fungi, offered an additional layer of comprehensive ecological insights beyond what tools like FUNGuild or FungalTraits can provide, highlighting the need for a dedicated PiCrust2‐like database tailored specifically for fungi. Our analysis also highlighted many unanswered questions. Can bacteria alone be allocated to these processes when many fungal pathways, particularly in endophytes, remain undocumented? How can we assign pathways to bacteria when bacteria reside inside fungi (Bonfante and Desirò 2017; Olsson et al. 2017; Deveau et al. 2018), or disentangle metabolic contributions during degradation when DNA and metabolic pathways from plants, fungi, and bacteria are intermingled? The role of horizontal gene transfer (HGT) during degradation is also unclear, despite its importance in amino acid biosynthesis, community development, and metabolic regulation, especially for bacteria, yet it is rarely studied in fungi.

These findings underscore the need for improved fungal sequencing and taxonomy, as many environmental fungi, particularly endophytes, remain uncharacterised. Expanding genetic mapping beyond model organisms (e.g., *S. cerevisiae* , *A. niger* , *A. fumigatus* or *Candida* sp.) is crucial to understanding fungal processes in natural environments. A better understanding of degradation pathways will enhance nutrient cycling knowledge, support biotechnological applications like bioremediation, inform kelp forest conservation and carbon flux modelling, and may highlight fungi's critical but greatly underexplored roles in microbial ecosystems. Fungi stand out as potential mediators (Fontaine et al. 2011; Vallet et al. 2018; Bahram and Netherway 2022) in these processes, and further research is needed to distinguish their precise contributions from those of bacteria.

## Author Contributions

**Anita K. Perkins:** conceptualization, methodology, writing – original draft, visualization, project administration, data curation, funding acquisition, formal analysis, software, investigation, validation. **Hans‐Peter Grossart:** conceptualization, writing – review and editing, supervision, methodology. **Keilor Rojas‐Jimenez:** data curation, writing – review and editing, software, formal analysis. **Alice Retter:** software, data curation, writing – review and editing, formal analysis. **Joanne M. Oakes:** conceptualization, methodology, writing – review and editing, supervision, resources.

## Conflicts of Interest

The authors declare no conflicts of interest.

## Supporting information


Data S1.

## Data Availability

All data relevant is in Supporting Information.
