# Supplementary material for: The Functional Role of Fungi and Bacteria in Sulfur Cycling During Kelp (Ecklonia Radiata) Degradation: Unconventional Use of PiCrust2
Source: Environ Microbiol Rep. 2025 Jul 24;17(4):e70140. doi: 10.1111/1758-2229.70140 (PMC12289537; doi:10.1111/1758-2229.70140)
Supplement: Supplementary file 1 — Data S1. [file EMI4-17-e70140-s001.zip › SI-Perkins-etal-Envi-Micr-Reports-2.docx]

The Functional Role of Fungi and Bacteria in Sulfur Cycling during Kelp (Ecklonia radiata) Degradation: Unconventional Use of PiCrust2

Anita K. Perkins^1,2,6^, Hans-Peter Grossart^3,4^, Keilor Rojas-Jimenez^5^, Alice Retter^3^, Joanne M. Oakes^6^

^1^Aquatic Botany and Microbial Ecology Research Group, HUN-REN Balaton Limnological Research Institute, 8237. Tihany, Hungary

^2^National Laboratory for Water Science and Water Security, HUN-REN Balaton Limnological Research Institute, 8237. Tihany, Hungary

^3^Leibniz Institute for Freshwater Ecology and Inland Fisheries (IGB), Experimental Limnology, 16775 Neuglobsow, Germany

^4^University of Potsdam, Institute of Biochemistry and Biology, Maulbeerallee 2, 14469 Potsdam, Germany

^5^Escuela de Biologia, Universidad de Costa Rica, 11501, San Jose, Costa Rica

^6^Centre for Coastal Biogeochemistry, Faculty of Science and Engineering, Southern Cross University, Lismore, 2480 NSW, Australia

Corresponding author: Anita Perkins (anita.perkins@blki.hu)

# Methods


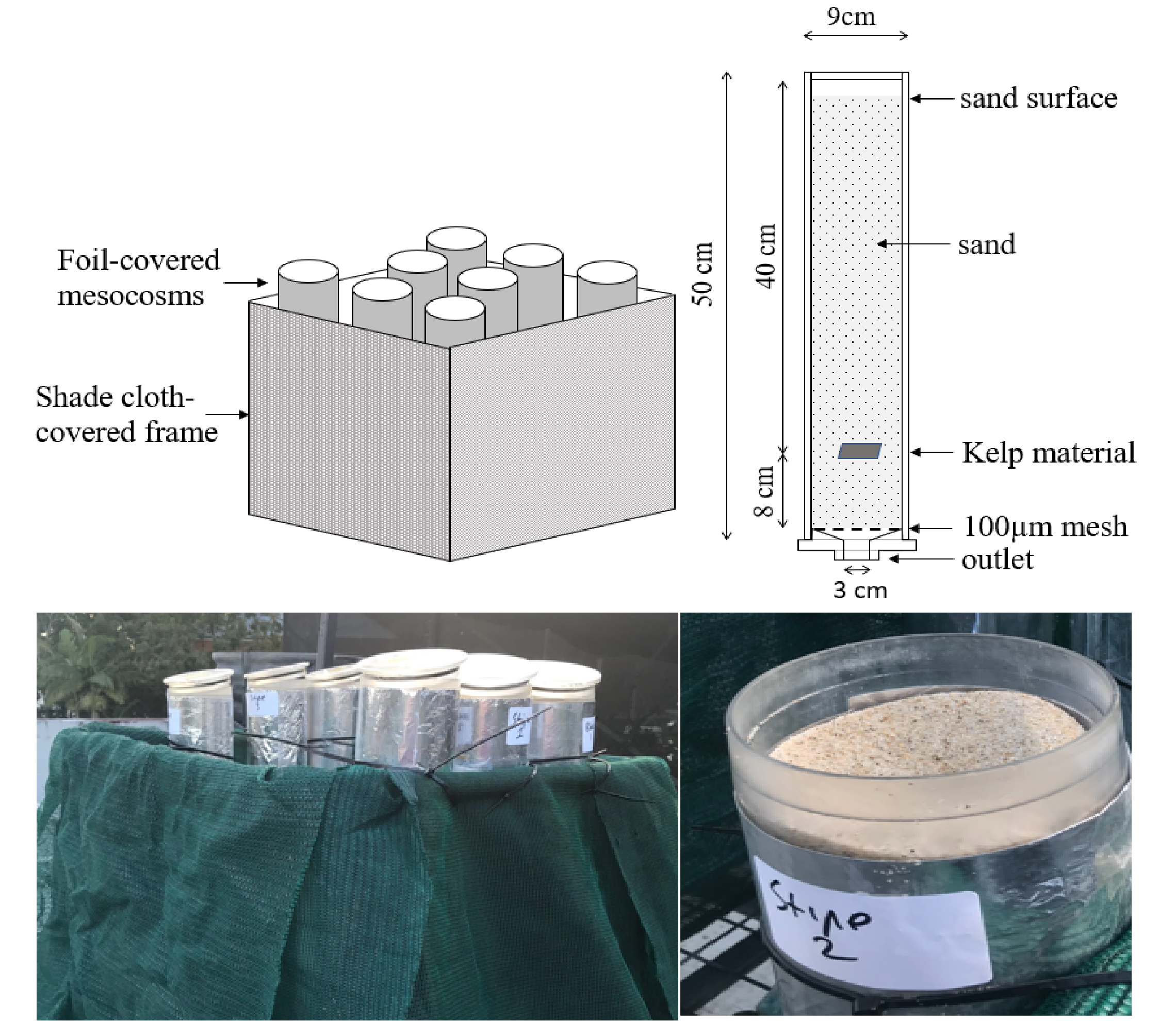


Figure S1 Mesocosms experimental setup

## Microbial analysis

Storing samples in 70% ethanol may not fully inhibit microbial activity and may lead to DNA degradation, potentially influencing the observed community composition, this approach was recommended by Australian Genome Research Facility Ltd., Australia (AGRF) following repeated attempts to sequence fungal communities from E. radiata. Despite multiple DNA extraction trials, we encountered persistent difficulties in obtaining high-quality fungal DNA. This was likely due to a combination of factors, including the presence of PCR inhibitors commonly found in marine macroalgal tissues (e.g., high levels of polysaccharides and polyphenols) and the overwhelming abundance of host DNA. Although fungal-specific primers were used, low fungal biomass may have hindered amplification efficiency relative to the host. As a result, we proceeded with samples below the optimal DNA concentration threshold, which yielded usable material for sequencing. This compromise was necessary to recover a detectable fungal signal and enable downstream community analysis. Samples surrounded with cool packs were sent to the AGRF via overnight delivery within one day of final sample collection.

DNA extraction was performed by AGRF using the DNeasy PowerSoil Pro Kit (QIAGEN), with an additional step of freeze-drying the kelp, followed by crushing with sterile steel balls on a TissueLyser II. Following extraction, primary analysis was conducted using MiSeq Control Software v3.1.0.13 and Real-Time Analysis (RTA) v1.18.54.4. For the bacterial amplicon library, the V3-V4 region of the 16S rRNA gene was amplified with universal primers 341F (CCTAYGGGRBGCASCAG) and 806R (GGACTACNNGGGTATCTAAT)^1^. Meanwhile, the fungal amplicon library was generated by amplifying the ITS1 region using the primers ITS1-F (5´CTTGGTCATTTAGAGGAAGTAA) and ITS2 (5´GCTGCGTTCTTCATCGATGC)^2^. First, the PCR products were cleaned using magnetic beads and visualized on a 2% SYBR E-gel (Thermo Fisher Scientific). A secondary PCR amplification was then performed using an Applied Biosystems 384 Veriti Thermal Cycler with the Platinum SuperFi II Master Mix (Life Technologies, Australia). For both primer sets, the PCR conditions were as follows: initial denaturation at 98 °C for 30 seconds, followed by 30 cycles of denaturation at 98 °C for 10 seconds, annealing at 60 °C for 10 seconds, and extension at 72 °C for 30 seconds, with a final extension at 72 °C for 5 minutes. The resulting amplicons were subsequently cleaned using Agencourt AMPure XP magnetic beads (Beckman Coulter), quantified using a Promega Quantifluor fluorometer, and normalized to 5 ng µL^-1^ prior to sequencing. An equimolar pool was then prepared, cleaned, and concentrated using magnetic beads, and fragment size was confirmed using a High-Sensitivity D1000 Tape on an Agilent 2200 TapeStation. The pool was diluted to 5 nM, and molarity was re-confirmed using a Qubit High Sensitivity dsDNA assay (Thermo Fisher Scientific). Finally, sequencing was conducted on an Illumina MiSeq platform (San Diego, CA, USA) using a V3, 600-cycle kit (2 × 300 bp paired-end) at AGRF.

# Results

Table S1 PICRUSt2-inferred fungal metabolic pathways identified across kelp blade, stipe, and associated sediment samples. Pathways are annotated by functional category, expected host organism (MetaCyc), fungal relevance, and biochemical roles (fermentation type, redox activity, sulfur and NAD involvement). Fold-change values indicate temporal shifts (start vs. end) and differential enrichment across tissue types and treatment conditions (e.g., blade/stipe vs. control).

Table S2 PERMANOVA and post hoc pairwise comparisons of bacterial community composition at ASV and class levels, based on Bray–Curtis dissimilarities. Each sheet corresponds to comparisons by tissue, time, or their interaction. PERMANOVA results include pseudo-F-statistics and raw p-values, while post hoc pairwise contrasts include raw and FDR-corrected p-values (Benjamini–Hochberg method) to account for multiple testing.

Table S3 PERMANOVA and post hoc pairwise comparisons of bacterial community composition at ASV and class levels, based on Bray–Curtis dissimilarities. Each sheet corresponds to comparisons by tissue, time, or their interaction. PERMANOVA results include pseudo-F-statistics and raw p-values, while post hoc pairwise contrasts include raw and FDR-corrected p-values (Benjamini–Hochberg method) to account for multiple testing.

Table S4 Summary of sequencing data for bacterial (16S rRNA) and fungal (ITS) amplicons. Tabs include total ASV tables (16S-All, ITS-All), quality- or taxonomy-filtered subsets (16S-Selected, ITS-Selected), and an overview of raw sequencing output per sample (Total-Raw seq. count).

Table S5 Summary of Kruskal–Wallis ANOVA test results based on PiCrust2 functional pathway predictions (n = 424 per group). The table displays mean and sum of ranks for each of the seven tissue and sand sample groups. The overall Chi-square test yielded a significant result (χ² = 622.21382, df = 6, p < 0.0001), indicating significant differences in predicted metabolic potential across the sample groups during Ecklonia radiata degradation.

| **Sample Group** | **Mean Rank** | **Sum Rank** |
| --- | --- | --- |
| Stipe Start | 828.02 | 351082 |
| Stipe End | 1362.28 | 577605.5 |
| Blade Start | 1071.49 | 454313.5 |
| Blade End | 1511.85 | 641023 |
| Control Sand | 1938.51 | 821929.5 |
| Blade Sand | 1830.18 | 775996.5 |
| Stipe Sand | 1849.17 | 784046 |

Table S6 Friedman ANOVA test and Dunn’s post-hoc pairwise comparisons for PiCrust2 metabolic functional prediction scores across sample groups (n = 424 per group). The Friedman test detected significant differences among groups (Chi-square = 1835.53, df = 6, p < 0.0001). Post-hoc comparisons using the Wilcoxon-Nemenyi-McDonald-Thompson test identified which specific pairs differed significantly, with all reported comparisons showing p < 0.0001.

| **Sample Group** | **Sum Rank Diff** | **Z** | **Prob** | **Sig** |
| --- | --- | --- | --- | --- |
| Stipe-start "Stipe-end" | -655 | 14.72499 | <0.0001 | 1 |
| Stipe-start "Blade-start" | -309 | 6.9466 | <0.0001 | 1 |
| Stipe-start "Blade-end" | -996.5 | 22.40222 | <0.0001 | 1 |
| Stipe-start "Control-2-sand" | -2149.5 | 48.32269 | <0.0001 | 1 |
| Stipe-start "Blade-2-sand" | -1514 | 34.03608 | <0.0001 | 1 |
| Stipe-start "Stipe-3-sand" | -1680.5 | 37.77915 | <0.0001 | 1 |
| Stipe-end "Blade-start" | 346 | 7.77839 | <0.0001 | 1 |
| Stipe-end "Blade-end" | -341.5 | 7.67723 | <0.0001 | 1 |
| Stipe-end "Control-2-sand" | -1494.5 | 33.5977 | <0.0001 | 1 |
| Stipe-end "Blade-2-sand" | -859 | 19.31109 | <0.0001 | 1 |
| Stipe-end "Stipe-3-sand" | -1025.5 | 23.05416 | <0.0001 | 1 |
| Blade-start "Blade-end" | -687.5 | 15.45562 | <0.0001 | 1 |
| Blade-start "Control-2-sand" | -1840.5 | 41.3761 | <0.0001 | 1 |
| Blade-start "Blade-2-sand" | -1205 | 27.08948 | <0.0001 | 1 |
| Blade-start "Stipe-3-sand" | -1371.5 | 30.83255 | <0.0001 | 1 |
| Blade-end "Control-2-sand" | -1153 | 25.92048 | <0.0001 | 1 |
| Blade-end "Blade-2-sand" | -517.5 | 11.63387 | <0.0001 | 1 |
| Blade-end "Stipe-3-sand" | -684 | 15.37694 | <0.0001 | 1 |
| Control-2-sand "Blade-2-sand" | 635.5 | 14.28661 | <0.0001 | 1 |
| Control-2-sand "Stipe-3-sand" | 469 | 10.54354 | <0.0001 | 1 |
| Blade-2-sand "Stipe-3-sand" | -166.5 | 3.74307 | 0.11391 | 0 |


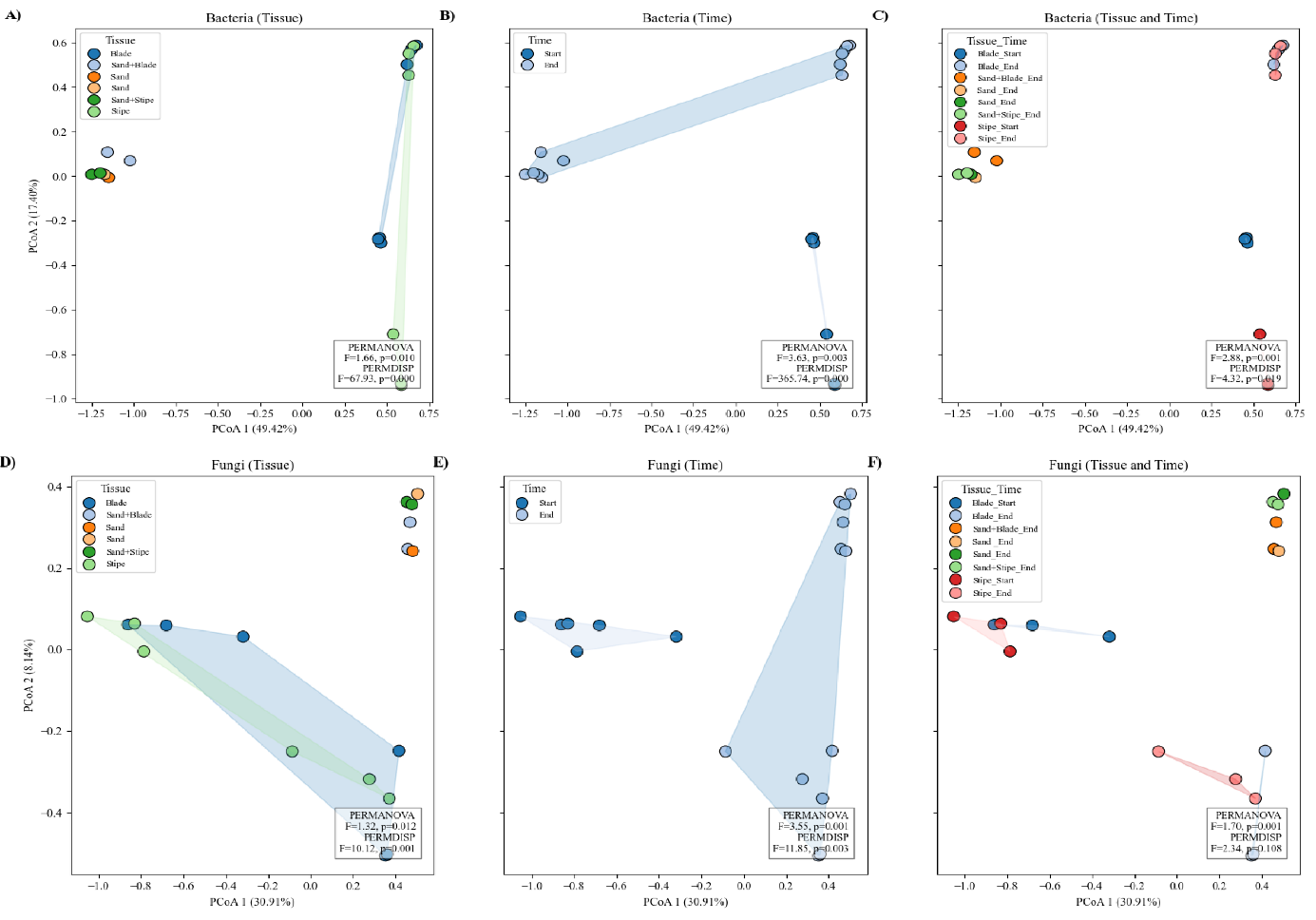


Figure S2 Principal Coordinate Analysis (PCoA) comparing the variation in bacterial amplicon sequence variance (ASV) (A–C) and fungal ASVs (D–F), where tissues (A and D), sampling times (B and E), and their interactions (C and F) are represented during *E. radiata* degradation. The ellipses highlight the grouping patterns based on significant factors. PERMDISP results indicate the homogeneity of multivariate dispersions, with the percentage of variation explained by each coordinate shown on the axis labels. Detailed statistics are in Supplementary Tables S2 and S3.


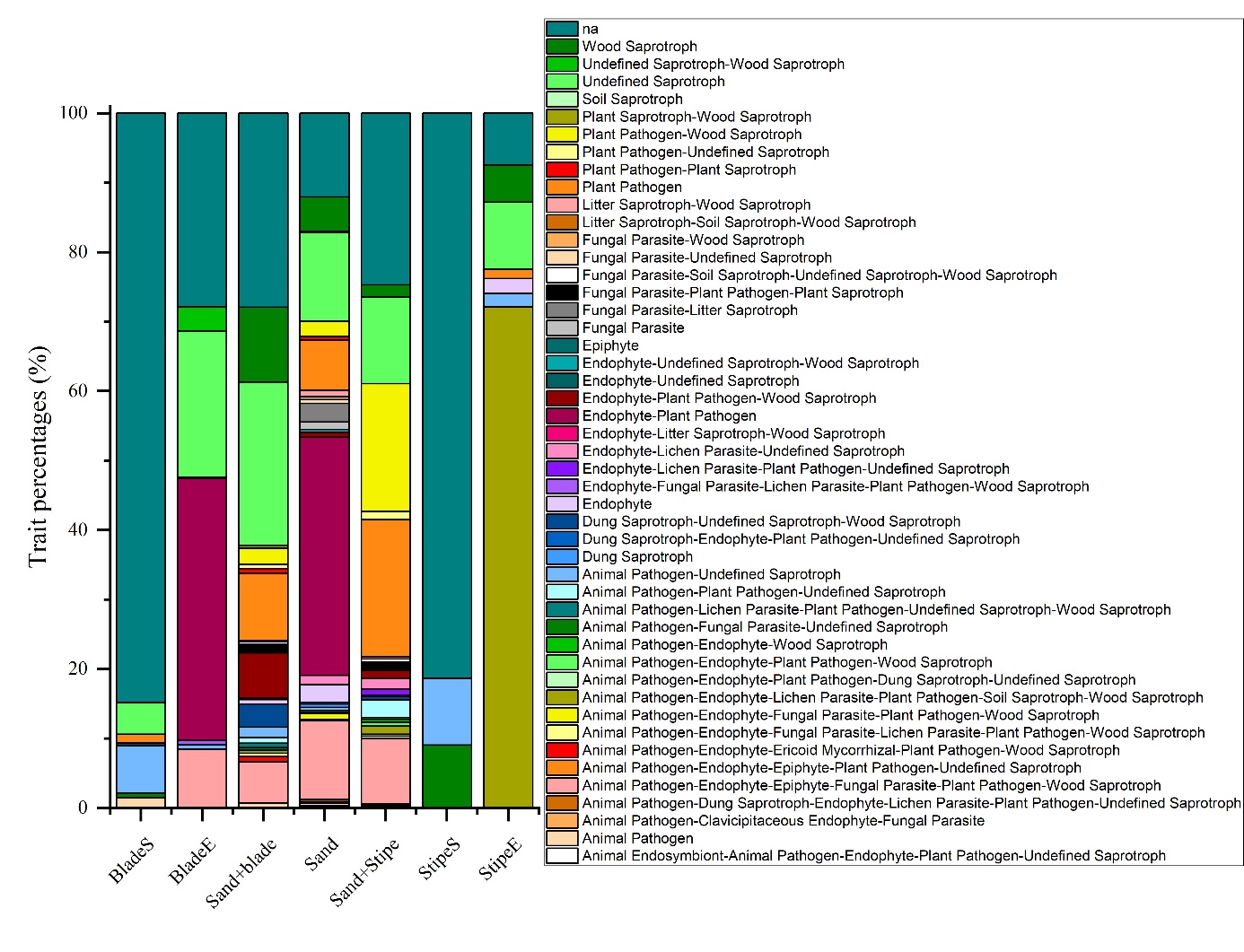


Figure S3 Ecological traits and guild assignments of fungal ASVs during Ecklonia radiata degradation based on the FUNGuild database, complete results of the secondary lifestyle classification from the traits template.


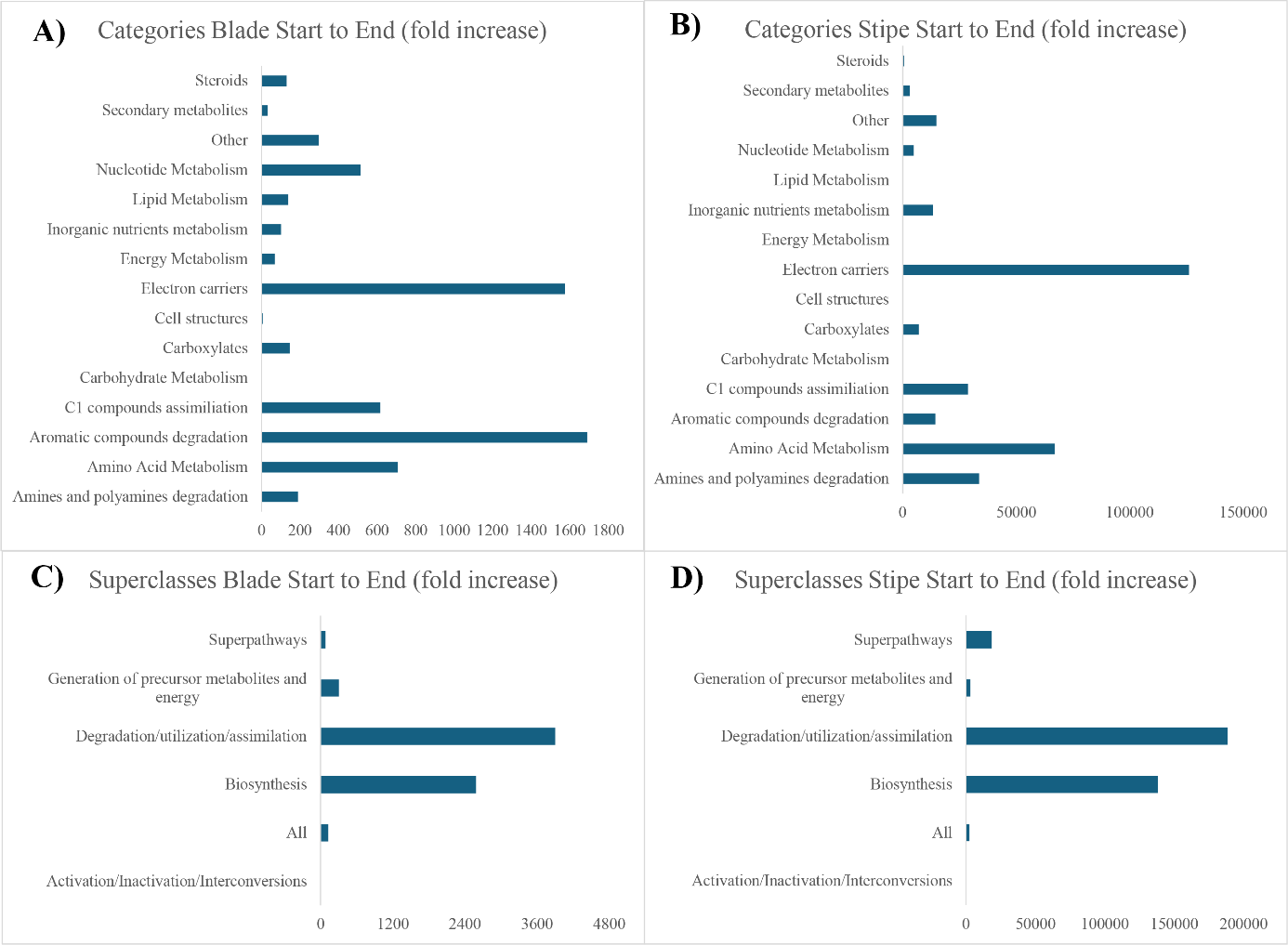


Figure S4 Summary of metabolic pathway categories and superclasses (fold increase) from Table S1, highlighting pathways with documented fungal involvement in the MetaCyc database or supported by literature. Panels (A and C) show “blade start” to “blade end” changes; while panels (B and D) show “stipe start” to “stipe end” changes.

Figure S5 PICRUSt2-inferred fungal metabolic pathways putatively involved in sulfur metabolism, identified through MetaCyc annotations and manual curation. Pathways are grouped by functional roles (e.g., biosynthesis, degradation, energy metabolism). Bars represent the mean relative abundance (± standard error) across “stipe start”, “stipe end”, and “sand+stipe” samples. The right panel shows log₂ fold changes for “stipe start” vs. “stipe end”, indicating temporal increases or decreases in pathway abundance potentially linked to sulfur processing.

Figure S6 PICRUSt2-inferred fungal metabolic pathways putatively involved in sulfur metabolism, identified through MetaCyc annotations and manual curation. Pathways are grouped by functional roles (e.g., biosynthesis, degradation, energy metabolism). Bars represent the mean relative abundance (± standard error) across “blade start”, “blade end”, and “Sand+Blade” samples. The right panel shows log₂ fold changes for “blade start” vs. “blade end”, indicating temporal increases or decreases in pathway abundance potentially linked to sulfur processing.

Figure S7 PICRUSt2-inferred fungal metabolic pathways putatively involved in sulfur metabolism, identified through MetaCyc annotations and manual curation. Pathways are grouped by functional roles (e.g., biosynthesis, degradation, energy metabolism). Bars represent the mean relative abundance (± standard error) across “blade”, “stipe”, and “control” samples. The right panel shows log₂ fold changes for “blade” vs. “control”, “stipe” vs. “control”, and “blade” vs. “stipe”, indicating differences in pathway abundance potentially linked to sulfur processing.

# References

1. Chen, K. H., Longley, R., Bonito, G. & Liao, H. L. A two-step PCR protocol enabling flexible primer choice and high sequencing yield for illumina miseq meta-barcoding. *Agronomy* vol. 11 Preprint at https://doi.org/10.3390/agronomy11071274 (2021).

2. Bellemain, E. *et al.* ITS as an environmental DNA barcode for fungi: An in silico approach reveals potential PCR biases. *BMC Microbiol* **10**, (2010).
