# Supplementary figures and images for: The Functional Role of Fungi and Bacteria in Sulfur Cycling During Kelp (Ecklonia Radiata) Degradation: Unconventional Use of PiCrust2
Source: Environ Microbiol Rep. 2025 Jul 24;17(4):e70140. doi: 10.1111/1758-2229.70140 (PMC12289537; doi:10.1111/1758-2229.70140)

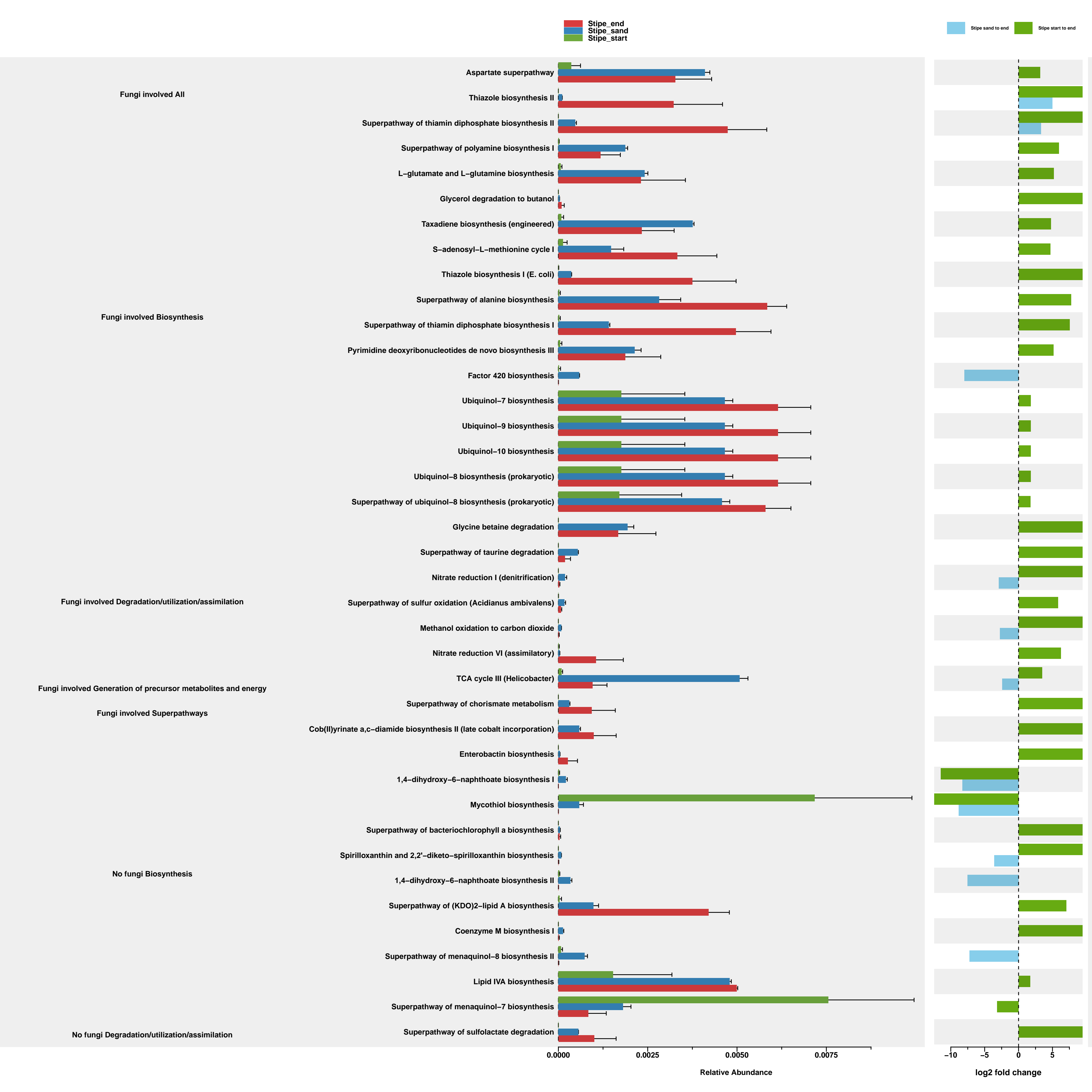

Supplement: Supplementary file 1 — Data S1. [file EMI4-17-e70140-s001.zip › FigS5.pdf]

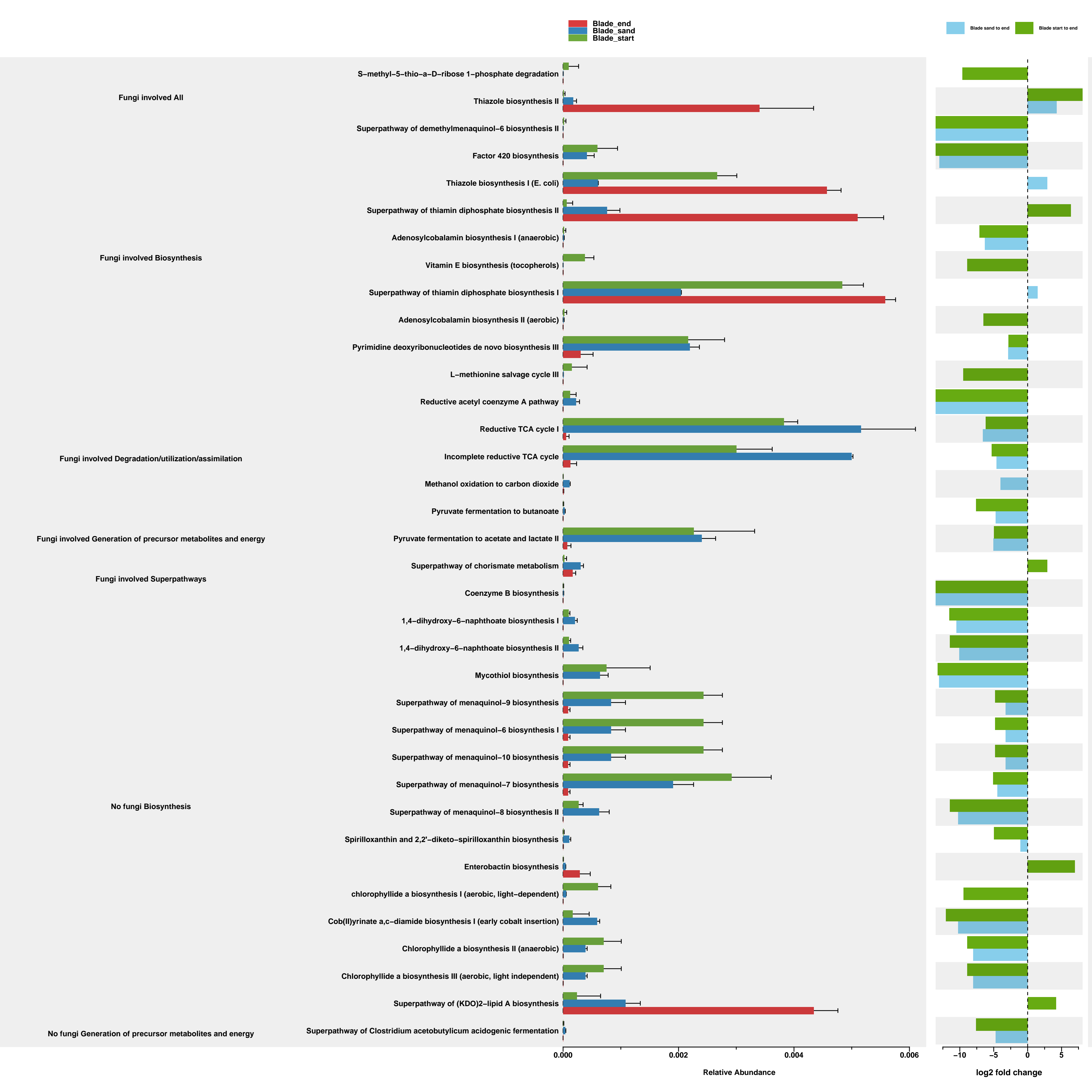

Supplement: Supplementary file 1 — Data S1. [file EMI4-17-e70140-s001.zip › FigS6.pdf]

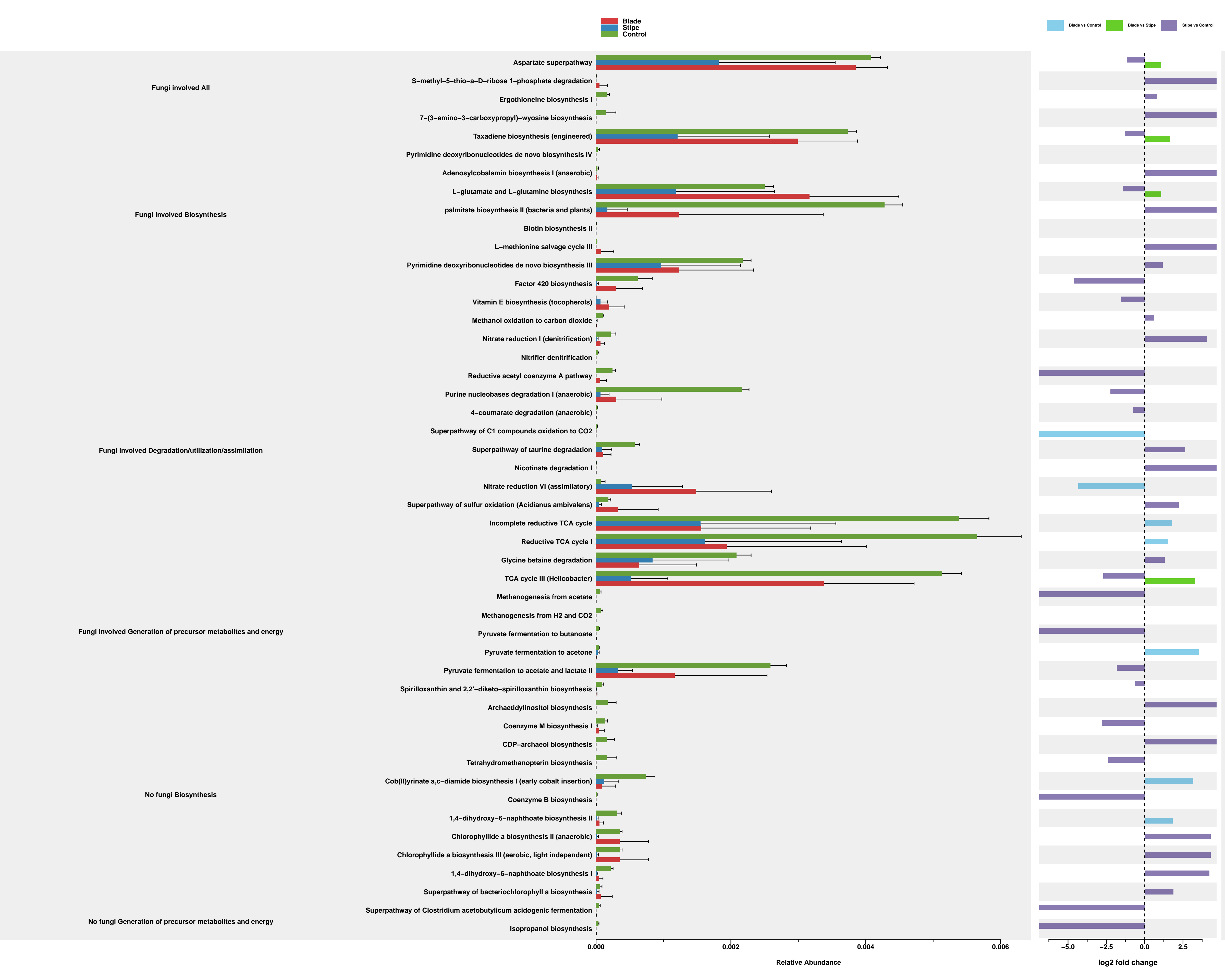

Supplement: Supplementary file 1 — Data S1. [file EMI4-17-e70140-s001.zip › FigS7.pdf]
